# An Integrated Computational Workflow for Discovering Alkaloid-Derived Ligands of Cyclin-Dependent Kinase 2

**DOI:** 10.3390/ph19081320

**Published:** 2026-08-21

**Authors:** Anh Tuan Do, Quoc Long Pham, Huong Thi Thu Phung, Minh Quan Pham

**Affiliations:** 1 Laboratory of Biophysics, Institute for Advanced Study in Technology, Ton Duc Thang University, Ho Chi Minh City 700000, Vietnam; doanhtuan@tdtu.edu.vn (A.T.D.); phamquoclong@tdtu.edu.vn (Q.L.P.); 2 Faculty of Pharmacy, Ton Duc Thang University, Ho Chi Minh City 700000, Vietnam; 3 NTT Hi-Tech Institute, Nguyen Tat Thanh University, Ho Chi Minh City 700000, Vietnam; ptthuong@ntt.edu.vn; 4 Institute of Chemistry, Vietnam Academy of Sciences and Technology, 18 Hoang Quoc Viet, Nghia Do, Hanoi 100000, Vietnam; 5 Faculty of Chemistry, Graduate University of Science and Technology, Vietnam Academy of Sciences and Technology, 18 Hoang Quoc Viet, Nghia Do, Hanoi 100000, Vietnam

**Keywords:** CDK2, cancer, alkaloid compounds, molecular docking, molecular dynamics, FPL

## Abstract

**Background/Objectives:** Cyclin-dependent kinase 2 (CDK2) is a key regulator of cell-cycle progression and a potential anticancer target. This study aimed to identify alkaloid-derived CDK2 ligands using an integrated computational workflow and to obtain preliminary evidence of their effects on cancer-cell viability. **Methods:** Molecular docking with mVina and fast pulling of ligand (FPL) simulations were benchmarked using 20 experimentally characterized CDK2 inhibitors. A library of 2692 PubChem-derived alkaloids was screened, followed by ADMET evaluation, 100 ns molecular dynamics simulations, and FPL-based relative-affinity re-ranking. The three prioritized compounds were evaluated in HepG2 and HGC-27 cells using an MTT assay after 48 h of exposure. **Results:** Docking and FPL showed correlations with experimental affinity data of R_Dock_ = 0.549 ± 0.180 and R_W_ = −0.676 ± 0.119, respectively. **CID 636885**, **CID 46184320**, **and CID 101691758** were prioritized for detailed evaluation. All three compounds reduced cell viability, with lower IC_50_ values observed in HepG2 cells than in HGC-27 cells. **CID 101691758** exhibited the highest growth-inhibitory activity among the tested compounds, with IC_50_ values of 15.37 ± 0.46 µg mL^−1^ in HepG2 cells and 52.64 ± 1.33 µg mL^−1^ in HGC-27 cells. **Conclusions:** The workflow identified three preliminary alkaloid hits, with **CID 101691758** showing the most favorable combined computational and cell-viability profile. However, the MTT assay does not establish direct CDK2 inhibition or kinase selectivity. Biochemical CDK2 inhibition, target-engagement, and kinase-panel studies are therefore required.

## 1. Introduction

Cancer remains one of the most significant global health concerns, characterized by its rapid spread and the lack of specific, highly effective treatment methods for various stages and types [1,2]. Historically, large-scale pandemics and diseases have caused millions of infections and deaths, leading to global economic crises and social upheaval. In the current era, the emergence of new variants and the limitations of existing therapies continue to pose a threat to human health on a global scale. Consequently, there is an urgent interest in discovering novel therapeutic agents to prevent the biological activity of key proteins associated with disease progression [2].

Cyclin-dependent kinase 2, commonly referred to as CDK2, is a key cell cycle regulatory enzyme that is fundamentally involved in controlling cellular proliferation and progression through the cell cycle [3]. Dysregulation and overexpression of CDK2 have been strongly associated with tumorigenesis, particularly in CCNE1-amplified ovarian and uterine cancers, as well as certain subsets of lung cancer [3,4]. While FDA-approved CDK4/6 inhibitors (e.g., palbociclib, ribociclib, and abemaciclib) have established clinical efficacy in HR+/HER2− breast cancer [5], the emergence of resistance to these therapies remains a major clinical challenge. Notably, this resistance is frequently driven by the compensatory activation of the CDK2/Cyclin E pathway, making CDK2 an essential therapeutic target to overcome or bypass CDK4/6 inhibitor resistance in breast cancer. Despite its profound therapeutic potential, no selective CDK2 inhibitor has yet achieved regulatory approval, primarily due to past challenges regarding narrow therapeutic windows, off-target toxicity, and insufficient selectivity [6]. Recent studies of selective CDK2 inhibitors, including INX-315 and BLU-222, have demonstrated antitumor activity in CCNE1-amplified and CDK4/6 inhibitor-resistant tumor models, while also highlighting the influence of molecular context and kinase selectivity on therapeutic response [7,8].

Natural products continue to play a vital role in drug discovery, with more than 60% of approved drugs being derived from or inspired by natural compounds [9]. Among them, alkaloids represent a promising class of bioactive molecules with diverse pharmacological activities. For example, alvocidib (flavopiridol), a flavone alkaloid, was one of the first multi-CDK inhibitors to enter clinical trials [10]. In addition, recent studies have demonstrated that alkaloids derived from natural sources, including marine organisms and medicinal plants, exhibit potent anticancer activities through CDK2 inhibition [9]. Marine fungi and bacteria, in particular, offer a sustainable and scalable platform for the production of structurally diverse bioactive compounds.

Computer-aided drug design (CADD) has emerged as an effective strategy for the rapid and reliable screening of extensive compound libraries to identify potential inhibitors. Its growing prominence is largely attributed to its capacity to markedly reduce both the time and financial burden associated with conventional drug discovery processes [10]. More recently, AI-assisted approaches have expanded the scope of CADD by enabling high-accuracy prediction of biomolecular interactions and generative design of drug candidates, with AI-derived molecules now progressing into clinical evaluation [11,12]. Among CADD methodologies, molecular docking is widely employed as an initial approach to predict ligand binding modes and estimate their affinities within the active site of target proteins [13]. Nevertheless, docking calculations inherently involve simplifying assumptions, which may limit their predictive accuracy. To overcome these limitations, atomistic simulation techniques such as molecular dynamics (MD) are employed to further refine docking results and evaluate the stability of protein–ligand complexes under conditions that resemble physiological environments [10]. In particular, accurate determination of the Gibbs free energy change (ΔG) upon binding is essential, as this parameter is directly correlated with the inhibition constant (K_i_) and experimentally observed binding affinities. MD simulations provide a time-resolved description of atomic motions, enabling detailed characterization of complex stability, conformational flexibility, and the persistence of key intermolecular interactions. Collectively, these insights are critical for a more reliable assessment of inhibitory potential [14].

Previous computational studies targeting CDK2 have employed pharmacophore-based virtual screening, structure- and ligand-based screening, molecular docking, and MD simulations to identify and prioritize potential inhibitors from natural-product and synthetic compound libraries [15,16,17]. Specifically, Lu et al. [15] performed virtual screening of Traditional Chinese Medicine compounds using molecular docking and molecular dynamics simulations to identify potential allosteric CDK2 inhibitors. Mahajan et al. [16] combined structure-based and ligand-based approaches for large-scale screening of chemically diverse compounds; whereas, Chagaleti et al. [17] employed molecular docking, molecular dynamics simulations, and binding free-energy analyses to investigate candidate CDK2 inhibitors. Although these studies demonstrated the utility of computational approaches for CDK2 inhibitor discovery, none incorporated a benchmarked FPL protocol for post-docking affinity ranking. Furthermore, the compound libraries investigated in previous studies differed substantially from the alkaloid-focused dataset examined in the present work. The principal methodological novelty of the present study lies in the use of FPL simulations as a post-docking validation and re-ranking step following the large-scale screening of 2692 alkaloids. Furthermore, four pulling speeds were systematically evaluated using 20 experimentally characterized CDK2-inhibitor complexes to identify a condition that provides an optimal balance between predictive performance and computational cost. The optimized workflow, integrating mVina docking (a modified version of AutoDock Vina) [18], ADMET (Absorption, Distribution, Metabolism, Excretion, and Toxicity) assessment, 100 ns MD simulations, and FPL calculations, was subsequently applied to prioritize alkaloid-derived ligands for CDK2. Initially, molecular docking simulations were performed to identify favorable ligand–protein binding conformations. Subsequently, ADMET evaluation was carried out to select compounds exhibiting suitable drug likeness, pharmacokinetic characteristics, and toxicity profiles. The resulting protein–ligand complexes were subsequently subjected to MD and FPL simulations to assess their dynamic stability and refine the docking-based ranking according to relative binding affinity. Notably, the FPL approach has demonstrated strong correlation with experimental data while requiring substantially lower computational resources compared to more demanding free-energy methods, with accuracy comparable to linear interaction energy and, in some cases, superior to end-point approaches [19]. Overall, the results suggest that three alkaloid compounds may represent preliminary CDK2-interacting hits and warrant further experimental validation and structural optimization.

## 2. Results

### 2.1. Molecular Docking Validation

Molecular docking, introduced in the 1980s, has since evolved into a widely applied computational approach, with numerous algorithms and software packages developed to explore protein–ligand interactions, particularly in drug discovery research [20]. The rapid performance and high computational efficiency of AutoDock Vina (Vina) [13] have contributed to its extensive use in predicting ligand–receptor interactions. It has been applied to tasks ranging from determining binding conformations of large substrates with protein targets to estimating the affinities of smaller entities such as peptides and proteins [21,22]. More recently, a modified variant, mVina, which incorporates empirically optimized parameters, has been reported to yield improved correlation with experimental data and enhanced docking accuracy.

**Figure 1:**
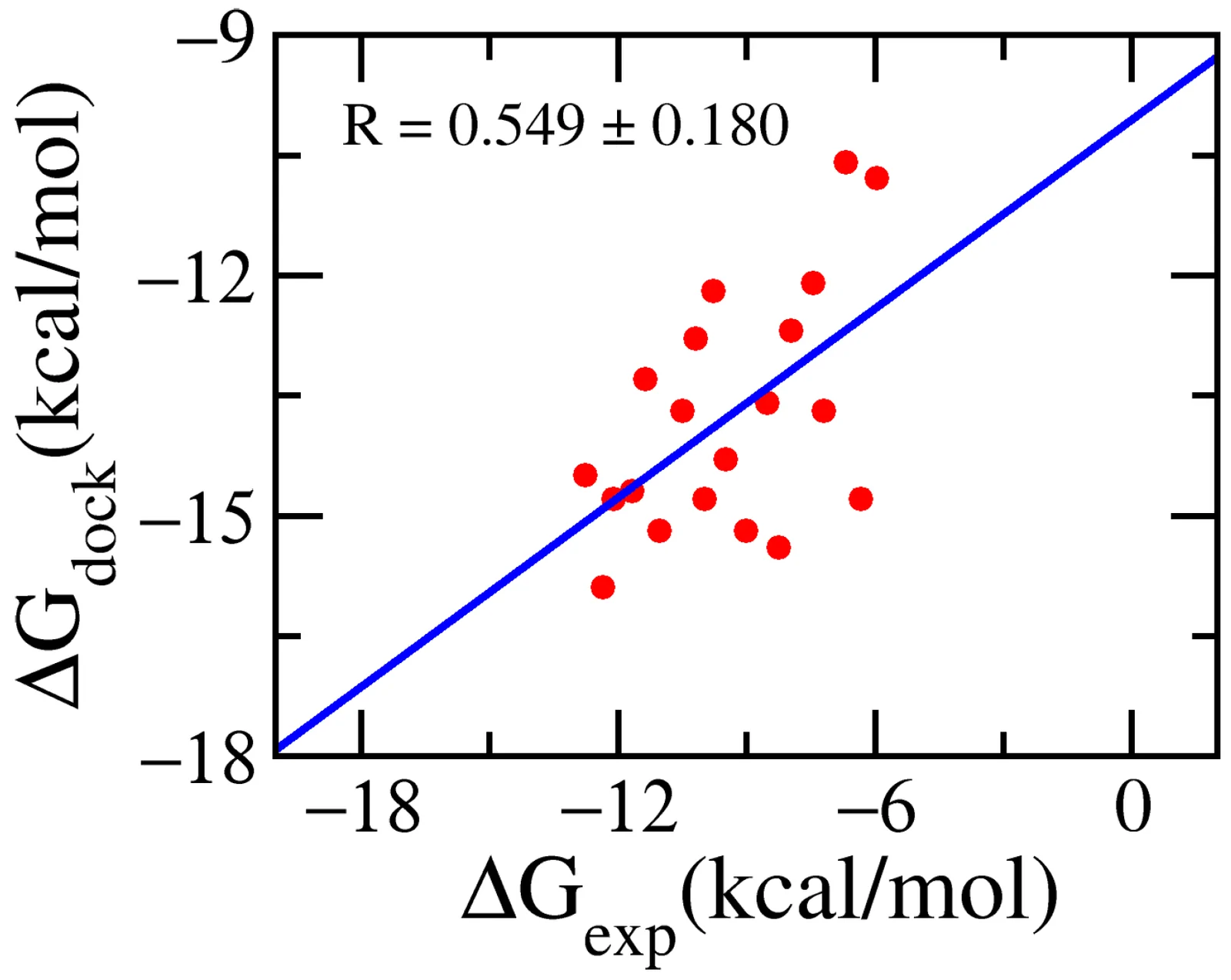
Correlation analysis between experimental (ΔG_exp_) and docking-calculated (ΔG_Dock_) binding free energies.

Twenty experimental structures of inhibitor-CDK2 complexes were retrieved, and their half-maximal inhibitory concentration (IC_50_) values were reported to differ significantly. These experimental data were subsequently converted into corresponding binding free energy values for comparative analysis. To assess the reliability of the computational protocol, twenty co-crystallized CDK2 structures with sequence identities exceeding 97% were obtained from the RCSB Protein Data Bank and subjected to re-docking experiments. The predicted binding affinities show reasonable agreement with experimental measurements, yielding a correlation coefficient of R_Dock_ = 0.549 ± 0.180 (cf. Table 1 and Figure 1). This result is comparable to the CDK2 cross-docking study of Duca et al., in which the distribution of correlations between docking scores and experimental log(IC_50_) values peaked at approximately R = 0.5 [23]. In addition, the discrepancy between the predicted and experimental results was evaluated using the root-mean-square error (RMSE), yielding a value of 4.68 ± 0.41 kcal mol^−1^. These results support the applicability of mVina for prioritizing ligand-binding affinities toward the CDK2 target.

**Figure 2:**
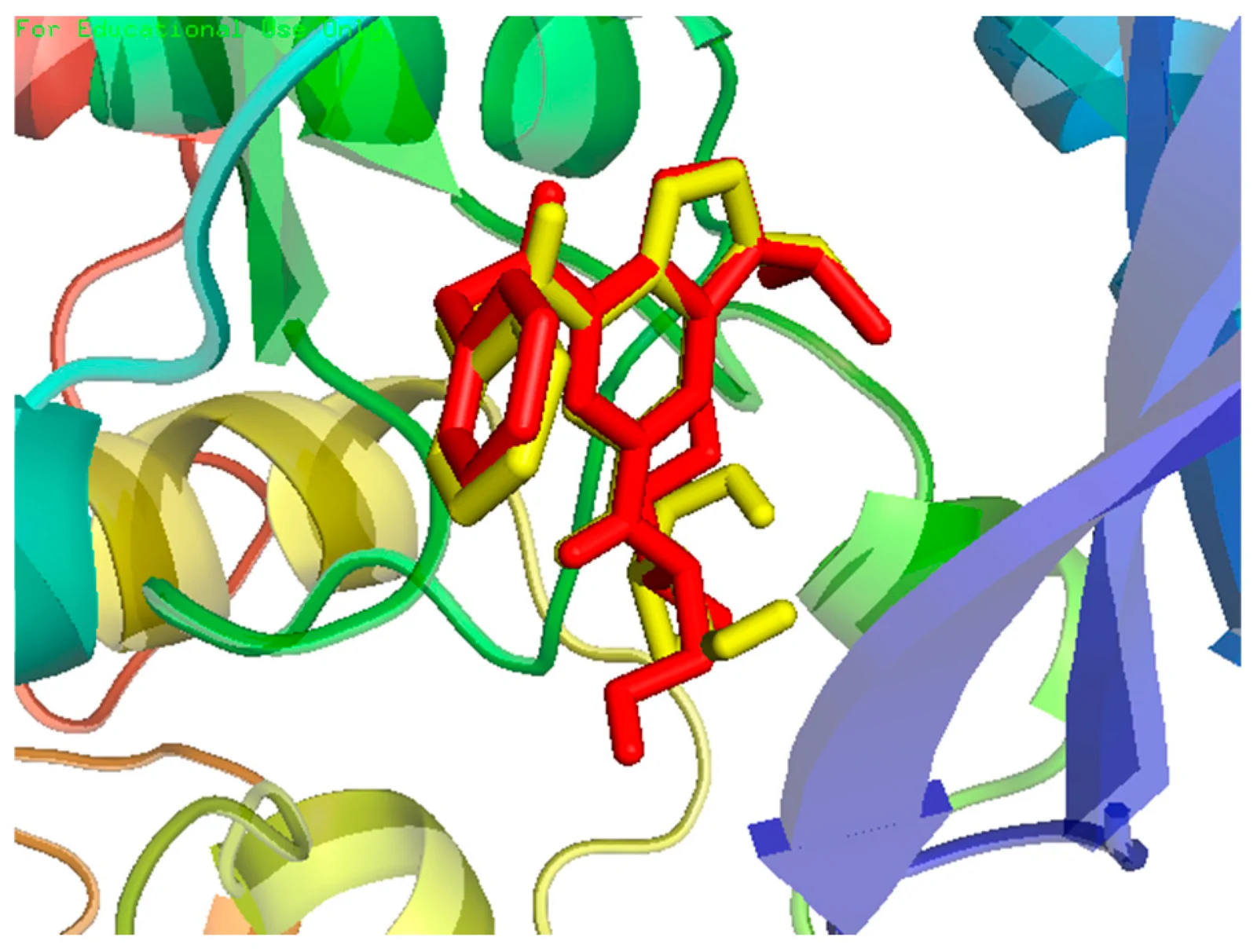
Superimpose of 3NS9 co-crystallized ligand (red) and its docking result (yellow).

Based on the above results, the binding affinities between the CDK2 protein and the 2692 alkaloid compounds were first predicted using mVina package. The CDK2 protein structure (PDB ID: 3NS9) was used as receptor, due to its 100% similarity to reference sequence, high resolution of 1.78 Å, and its co-crystallized ligand is similar to roscovitine and dinaciclib. The native ligand (BS-194) was redocked in the binding site of CDK2 protein. The top-ranked docking pose of BS-194 was aligned with the co-crystallized ligand to assess the reliability of the docking procedure. According to the criteria proposed by Gowthaman et al. [24], a docking methodology is considered as reliable when the root-mean-square deviation (RMSD) between the predicted binding conformation and the experimentally determined structure is less than 2.0 Å. In this study, the calculated RMSD was 0.589 Å, indicating good reproducibility of the docking approach (Figure 2).

**Figure 3:**
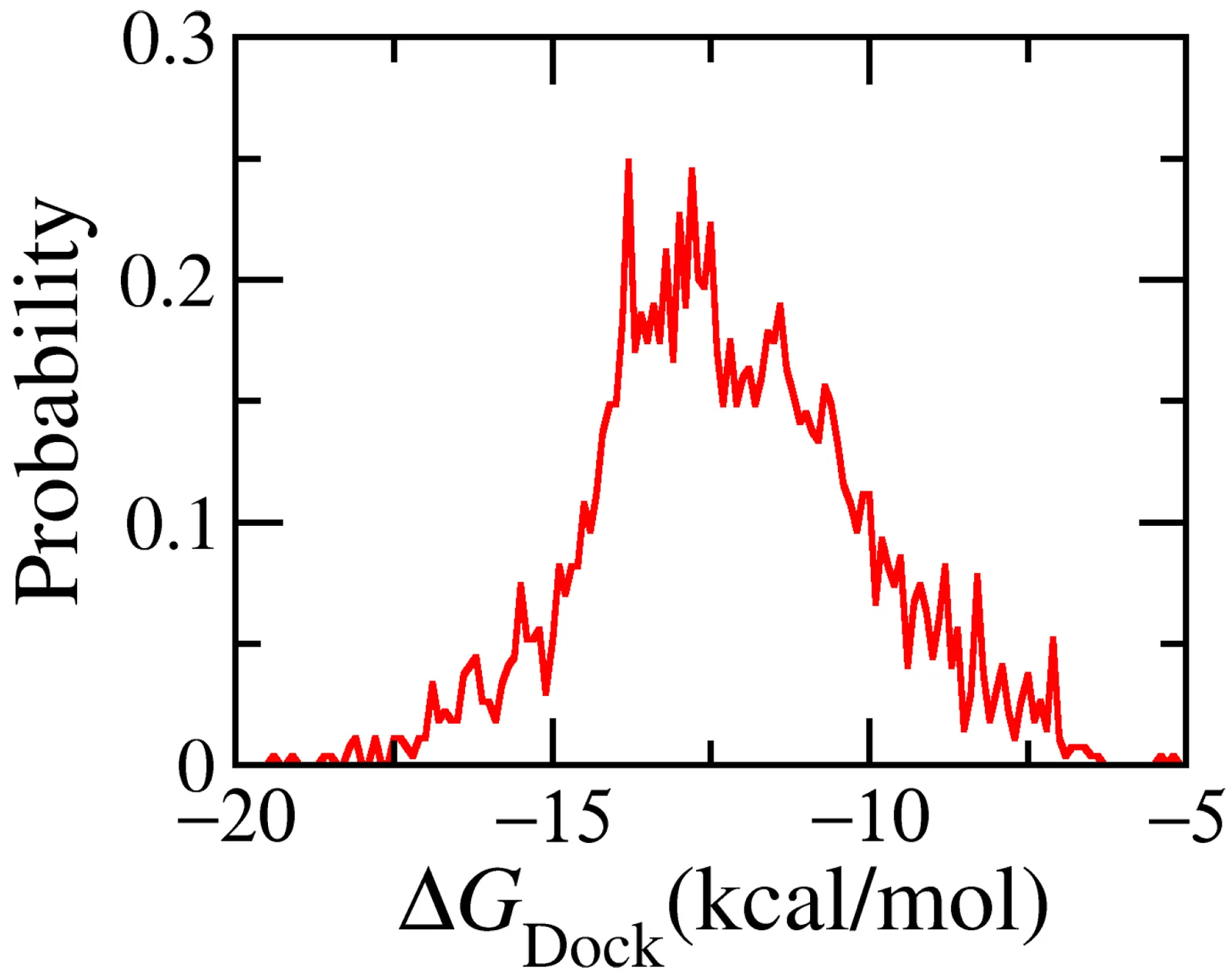
Distribution of the docking energy between 2692 alkaloid compounds targeting CDK2 protein.

The predicted binding free energies of the alkaloid compounds varied from −5.2 to −19.4 kcal mol^−1^, with a median value of −12.22 kcal mol^−1^ and a standard deviation of 2.12 kcal mol^−1^. The distribution pattern of the docking scores is illustrated in Figure 3. Based on the docking scores, the 26 highest-ranked compounds, representing approximately 1% of the screened library, were selected as docking candidates for subsequent ADMET evaluation.

### 2.2. ADMET Evaluation of Docking-Prioritized Compounds

Lipinski’s Rule of Five was employed to assess the drug-likeness characteristics and potential oral bioavailability of the 26 highest-ranked docked compounds, and the predicted ADMET parameters are summarized in Table 2.

**Figure 4:**
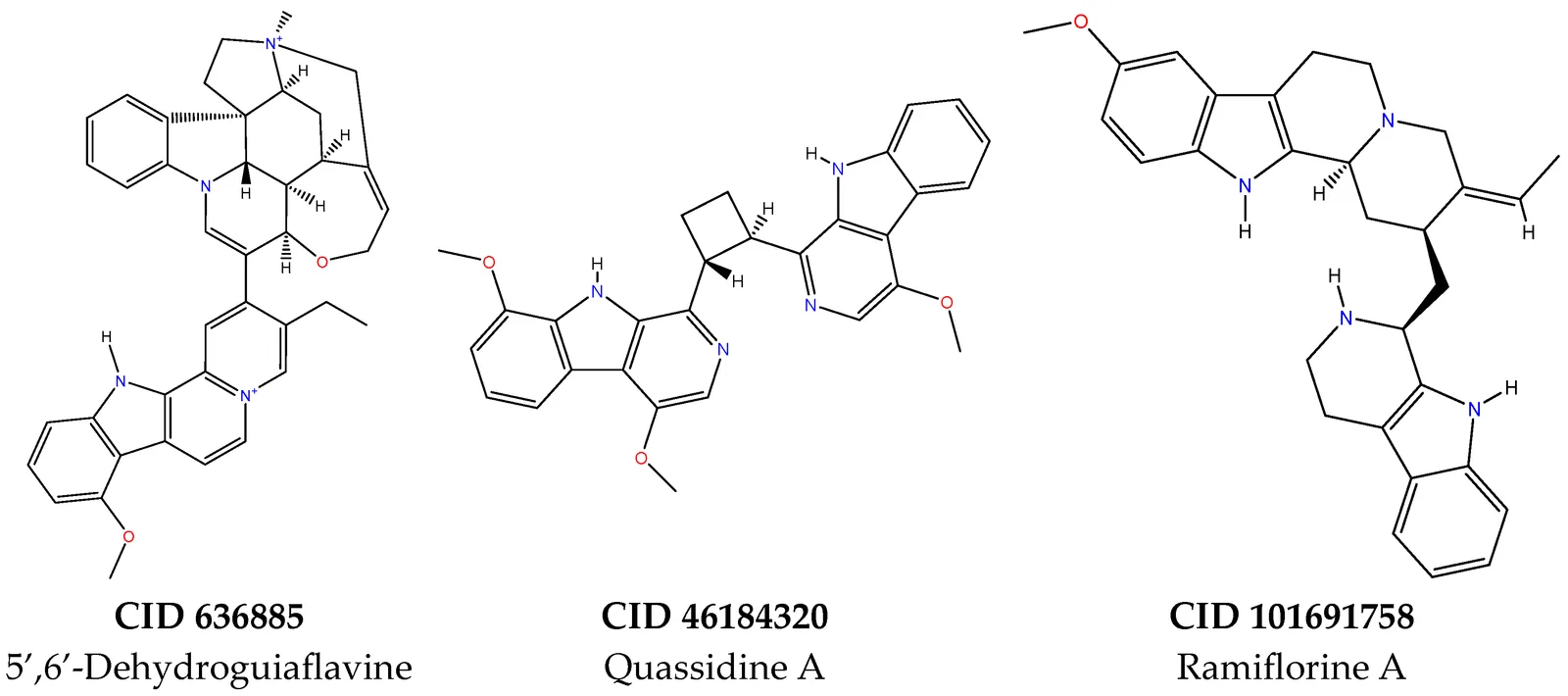
Chemical structures of the three alkaloid candidates. CID 636885 (5′,6′-dehydroguiaflavine), CID 46184320 (quassidine A), CID 101691758 (ramiflorine A).

In terms of safety, Ames test predictions indicate that **CID 636885**, **CID 46184320**, and **CID 101691758** are all non-mutagenic, satisfying a key requirement for further development. Analysis of physicochemical descriptors shows that their alogP values (1.69–4.97) fall within an acceptable range, suggesting a favorable balance between solubility and membrane permeability. Specifically, **CID 636885** exhibits lower lipophilicity (alogP = 1.69), which correlates with good aqueous solubility (134.5 mg L^−1^); whereas, **CID 46184320** (alogP = 4.32) and **CID 101691758** (alogP = 4.97) display higher lipophilicity, potentially enhancing interactions within the hydrophobic ATP-binding pocket of CDK2 while maintaining adequate solubility (155.5 and 113.4 mg L^−1^, respectively). Overall, the integration of docking results with ADMET filtering identifies these three compounds as three top-ranking candidates, combining high binding affinity with suitable pharmacokinetic and safety profiles for subsequent molecular dynamics investigations. The chemical structures of three top-lead candidates are shown in Figure 4.

### 2.3. Binding Conformation Analysis from Molecular Docking

**Figure 5:**
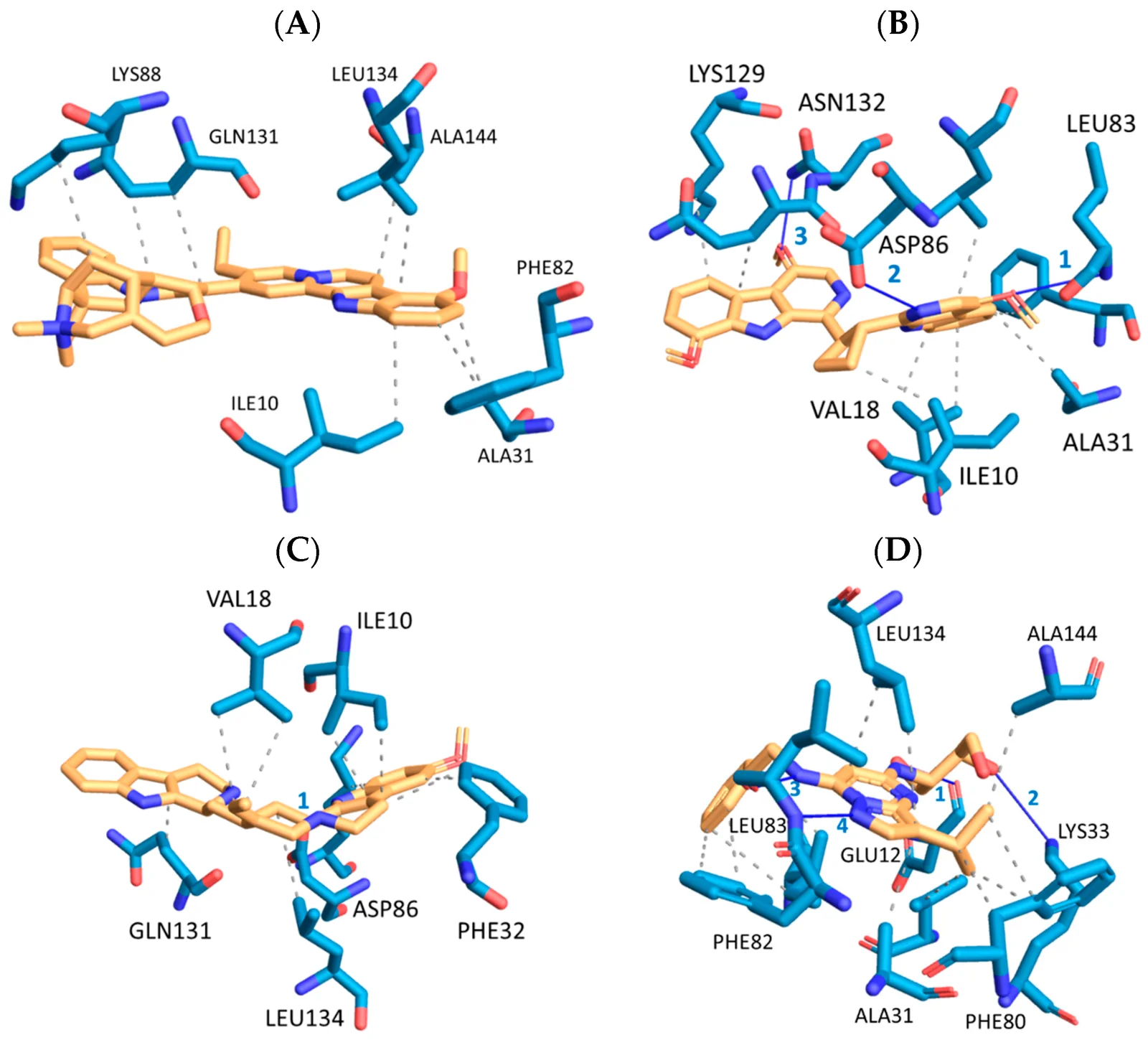
The binding pose of three prioritized compounds and native ligand on CDK2 obtained *via* molecular docking simulations. Hydrogen bond—navy line; hydrophobic interaction—black dashed line; π-stacking—green dashed line. (**A**) CID 636885; (**B**) CID 46184320; (**C**) CID 101691758; (**D**) BS-194. The interaction diagrams were generated using the Protein Ligand Interaction Profiler (PLIP) web tool.

To characterize the predicted interactions of the three prioritized compounds with CDK2, their docking poses within the binding pocket were analyzed. The predicted interaction patterns between CDK2 and the three top candidates are presented in Figure 5.

The co-crystallized ligand of 3NS9 establishes four hydrogen bonds with Glu12, Lys33, Phe82, and Leu83, accompanied by hydrophobic contacts with Ala31, Phe80, Leu134, and Ala144. Notably, Glu81, Leu83 and Asp86 constitute key residues within the ATP-binding pocket of type I CDK2 inhibitors, which compete directly with ATP [16]. Supportive of this binding mode, the ligand’s heterocyclic nitrogen atoms typically engage in multiple H-bonding interactions within the active site. By emulating the exocyclic amine of the ATP adenyl moiety, these bonds serve to facilitate robust anchoring of the inhibitor. Among the screened compounds, **CID 636885** exhibited the most favorable docking score (−19.4 kcal mol^−1^); however, it did not form any hydrogen bonds with surrounding residues. Instead, its binding is dominated by hydrophobic interactions involving Ile10, Ala31, Phe82, Lys88, Gln131, Leu134, and Ala144. This interaction pattern suggests a binding mode primarily driven by van der Waals and nonpolar contacts rather than specific directional interactions. Despite this limitation, the ligand adopts a suitable orientation within the ATP-binding pocket and demonstrates favorable ADMET and drug-likeness profiles, justifying its selection for subsequent molecular dynamics simulations. In contrast, **CID 46184320** forms three hydrogen bonds with Leu83, Asp86, and Asn132, indicating a stronger contribution of polar interactions to binding stability. Additional van der Waals contacts with Ile10, Val18, Ala31, and Lys129 further reinforce ligand accommodation within the pocket. Similarly, **CID 101691758** establishes a single hydrogen bond with Asp86 and is stabilized by an extensive network of hydrophobic interactions involving Ile10, Val18, Phe32, Gln131, and Leu134.

### 2.4. Validation and Optimization of the FPL Protocol

Despite the favorable correlation between mVina predictions and experimental data, molecular docking inherently involves several simplifying assumptions to improve computational efficiency. These include the use of a rigid receptor model, restricted sampling of ligand conformations, united-atom representations, and implicit solvent treatments [25]. These approximations may reduce the predictive reliability of the obtained results. Consequently, additional validation using more robust and computationally intensive methods is necessary to investigate the dynamic properties and stability of protein–ligand complexes under physiological environments. In the present study, FPL simulations were employed to further verify the docking results, as this approach has been shown to generate outcomes in good agreement with experimental data while retaining reasonable computational efficiency. Although the accuracy of FPL is generally lower than that of the free energy perturbation (FEP) approach, it requires substantially fewer computational resources [26]. Furthermore, unlike end-point free energy methods, FPL offers additional insight into the ligand unbinding pathway from the enzyme active site. In this research, the performance of the FPL protocol was initially evaluated using 20 experimentally characterized CDK2–inhibitor complexes.

The correlations between rupture force (F_max_) and experimental binding free energy (ΔG_exp_), as well as between pulling work (W) and ΔG_exp_ at different pulling speeds, are summarized in the Supporting Information (Figures S1–S8). In addition, the graph of F_max_ and W in the time revolution are provided in Tables S1–S4. In general, the computed metrics exhibit good agreement with the corresponding experimental data, supporting the reliability of the FPL approach employed in this study. Among the investigated pulling speeds, the strongest correlations were observed at k = 0.0005 nm ps^−1^, with Pearson correlation coefficients of R_F_ = −0.680 and R_W_ = −0.681. The second-best performance was obtained at k = 0.005 nm ps^−1^, yielding R_F_ = −0.652 and R_W_ = −0.676. Although the lowest pulling speed (k = 0.0005 nm ps^−1^) provides slightly higher correlation coefficients, it requires a considerably longer simulation duration, approximately tenfold greater than that of k = 0.005 nm ps^−1^ (7000 ps versus 700 ps). Given the marginal statistical difference in Pearson correlation coefficients between these two conditions, the use of a pulling speed of k = 0.005 nm ps^−1^ is considered optimal, offering a favorable balance between computational efficiency and predictive accuracy for the FPL methodology in this work. In particular, the obtained FPL results of pulling work are shown in Table 3 and the results of maximum pulling force (F_max_) are presented in Table S5.

**Figure 6:**
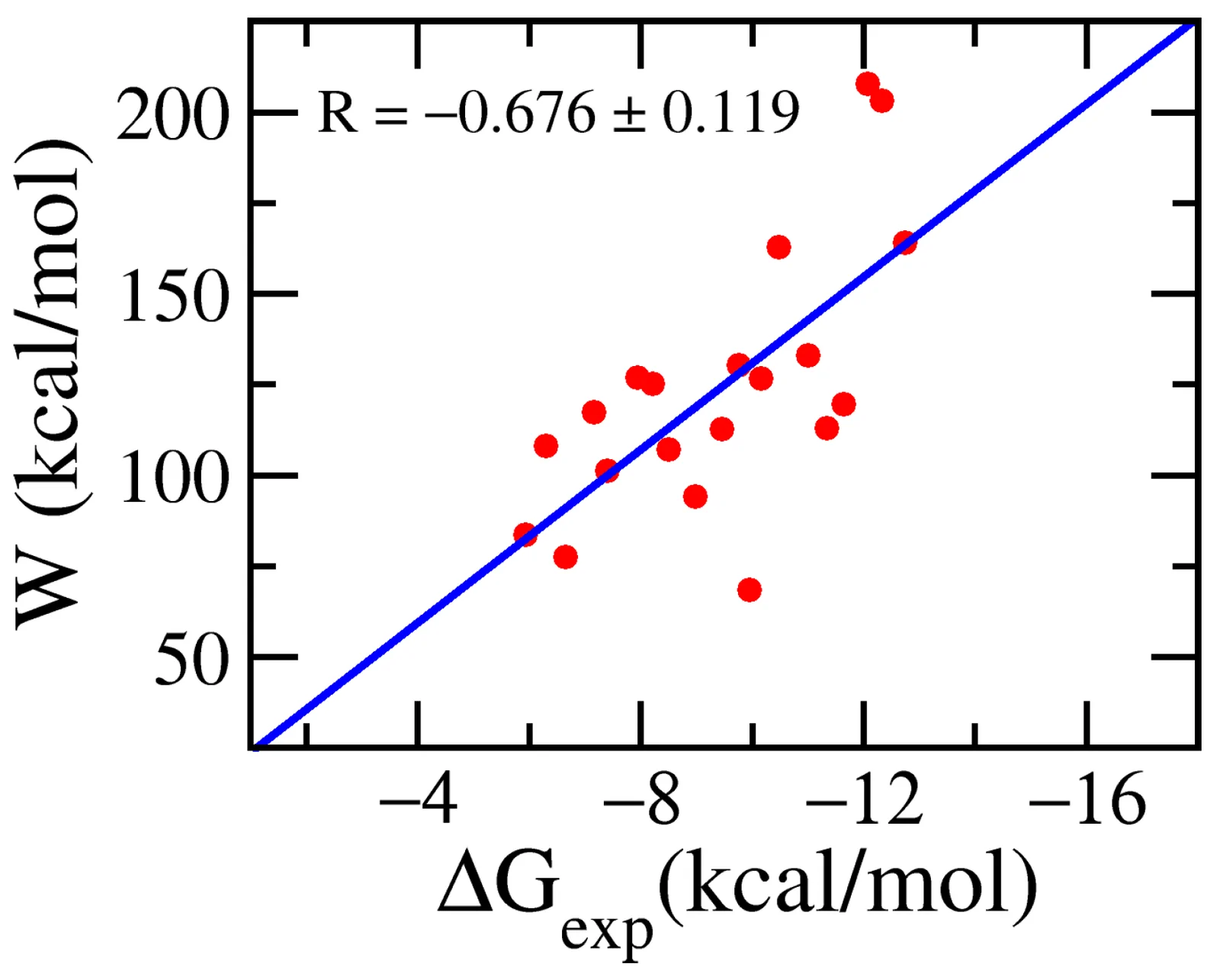
Correlation of W and ΔG_exp_ values (pulling speed of k = 0.005 nm ps^−1^).

Although the difference between R_F_ and R_W_ is quite small (−0.652 and −0.676, respectively), the pulling work was selected to rank the ligand-binding affinity to CDK2 (Figure 6). Accordingly, the predicted binding free energy ${\Delta G}_{FPL}^{Pre}$ can be estimated from the pulling work through linear regression analysis according to the following equation: ${∆G}_{FPL}^{Pre}=-0.084\times W+1.006$, where W is the pulling work. The accuracy of the FPL-based predictions was assessed using linear regression in combination with RMSE analysis, yielding an RMSE_W_ = 2.22 ± 0.36 kcal mol^−1^. The relatively low RMSE indicates that the FPL approach is capable of reliably distinguishing ligands with comparable binding affinities. Overall, these findings demonstrate that FPL simulations provide an effective and sufficiently accurate protocol for approach for ranking ligand-binding affinities toward CDK2.

### 2.5. FPL Simulations of Top-Ranking Compounds

**Figure 7:**
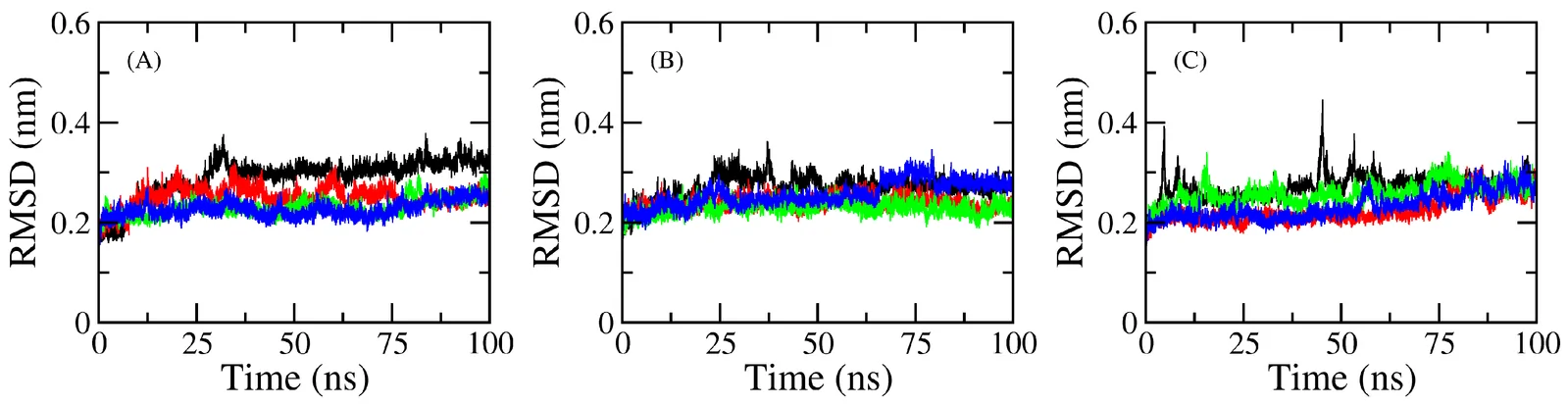
Non-hydrogen atoms RMSD of CDK2 + inhibitor complexes. (**A**) CID 636885; (**B**) CID 46184320; (**C**) CID 101691758.

FPL simulations were subsequently employed to re-rank the three docking-prioritized compounds according to their relative binding affinities toward CDK2. The resulting CDK2–inhibitor complexes were solvated in a box containing water molecules and counterions, and oriented such that the ligand unbinding pathway was aligned along the *Z*-axis. The systems were first equilibrated through conventional MD simulations, reaching stability after approximately 15 ns, as indicated by RMSD values converging around 0.2 nm (Figure 7).

To characterize the dissociation process, four independent FPL trajectories were generated for each complex. The resulting force–displacement profiles can be divided into three distinct phases: an initial rapid increase in force up to a maximum value (F_max_), followed by a decrease toward zero, and finally a region where the force fluctuates around zero (cf. Table S6 of the SI file). These features suggest the presence of two transition states during ligand unbinding. The first corresponds to the point at which the pulling force reaches F_max_, marking the onset of ligand detachment from the binding pocket, while the second is associated with the complete loss of non-bonded interactions between the ligand and the protein. The separation between these two transition states is approximately 0.9 nm, consistent with the typical range of non-covalent interactions.

Based on the FPL simulations, the average rupture forces and average pulling work values were distributed within the ranges of 654.1 ± 104.9 to 795.6 ± 78.7 pN and 73.6 ± 11.7 to 89.2 ± 8.8 kcal mol^−1^, respectively. The predicted ${IC}_{50}^{Pre}$ thus calculated via formula ${IC}_{50}^{Pre}$ = $e^{\left({\Delta G}_{FPL}^{Pre}/RT\right)}$, where T = 310 K is the absolute temperature and R = 1.987 cal mol^−1^ K^−2^ is gas constant, with an approximation that IC_50_ equals the inhibition constant K_i_ under specific kinetic and assay conditions. The acquired results were shown in Table 4.

The predicted metrics ${\Delta G}_{FPL}^{Pre}$ ranged from −5.17 to −6.48 kcal mol^−1^, corresponding to the estimated ${IC}_{50}^{Pre}$ values of 26.7 to 224.0 µM. Overall, these results suggest that the three top candidates possess potential inhibitory activity against CDK2 protein. Nevertheless, additional experimental investigations are required to confirm these computational predictions.

### 2.6. MD-Refined Binding Conformation Analysis

**Figure 8:**
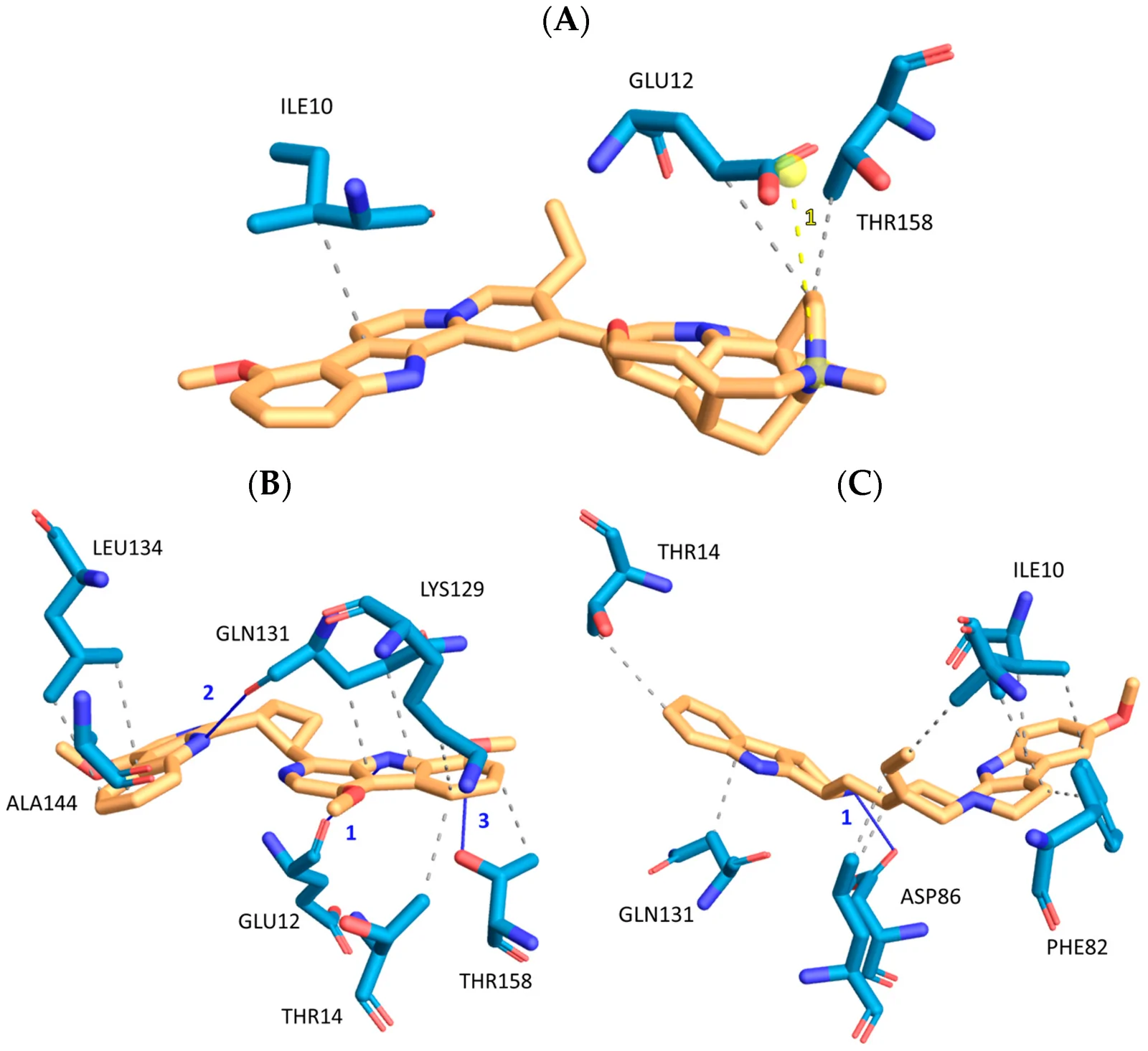
Binding conformations of the prioritized compounds within CDK2 following refinement by MD simulations. Hydrogen bond—navy line; hydrophobic interaction—black dashed line; π-stacking—green dashed line. (**A**) CID 636885; (**B**) CID 46184320; (**C**) CID 101691758. The interaction diagrams were generated using the PLIP web tool.

MD simulations also confirmed the occurrence of conformational changes during the binding process. The hydrogen bonds and hydrophobic interactions that were lost or newly formed before and after the simulations are illustrated in Figure 8.

For **CID 636885**, this compound maintained interactions with several key residues, including Ile10, Phe82, Gln131, and Leu134, while forming a new contact with Lys129. However, it lost its initial weak interactions with Ala31, Lys88, and Ala144. The persistent reliance on weak non-polar interactions without the establishment of stable hydrogen bonds (H-bonds) suggests that while **CID 636885** occupies the binding site, its long-term stability and specific inhibitory efficacy might be limited. In contrast, **CID 46184320** underwent substantial structural reorientation. This compound established three entirely new H-bonds with Thr14, Gln131, and Tyr159. This dynamic shift was accompanied by the retention of a hydrophobic contact with Val18 and the formation of new van der Waals interactions with Lys33, Leu134, Ala144, and Thr158. Notably, **CID 101691758** demonstrated the most stable binding behavior among the three investigated compounds during MD simulations. It successfully retained its initial crucial H-bond with Asp86, a key residue in the ATP-binding pocket. Furthermore, the compound established an additional H-bond at Ile10, enhancing its electrostatic anchoring. This strengthened polar network is comprehensively supported by a stable hydrophobic array involving Ala31, Phe82, Gln131, and Leu134, with Ala31 and Phe82 emerging as newly formed stable contacts compared to the static docking phase.

**Figure 9:**
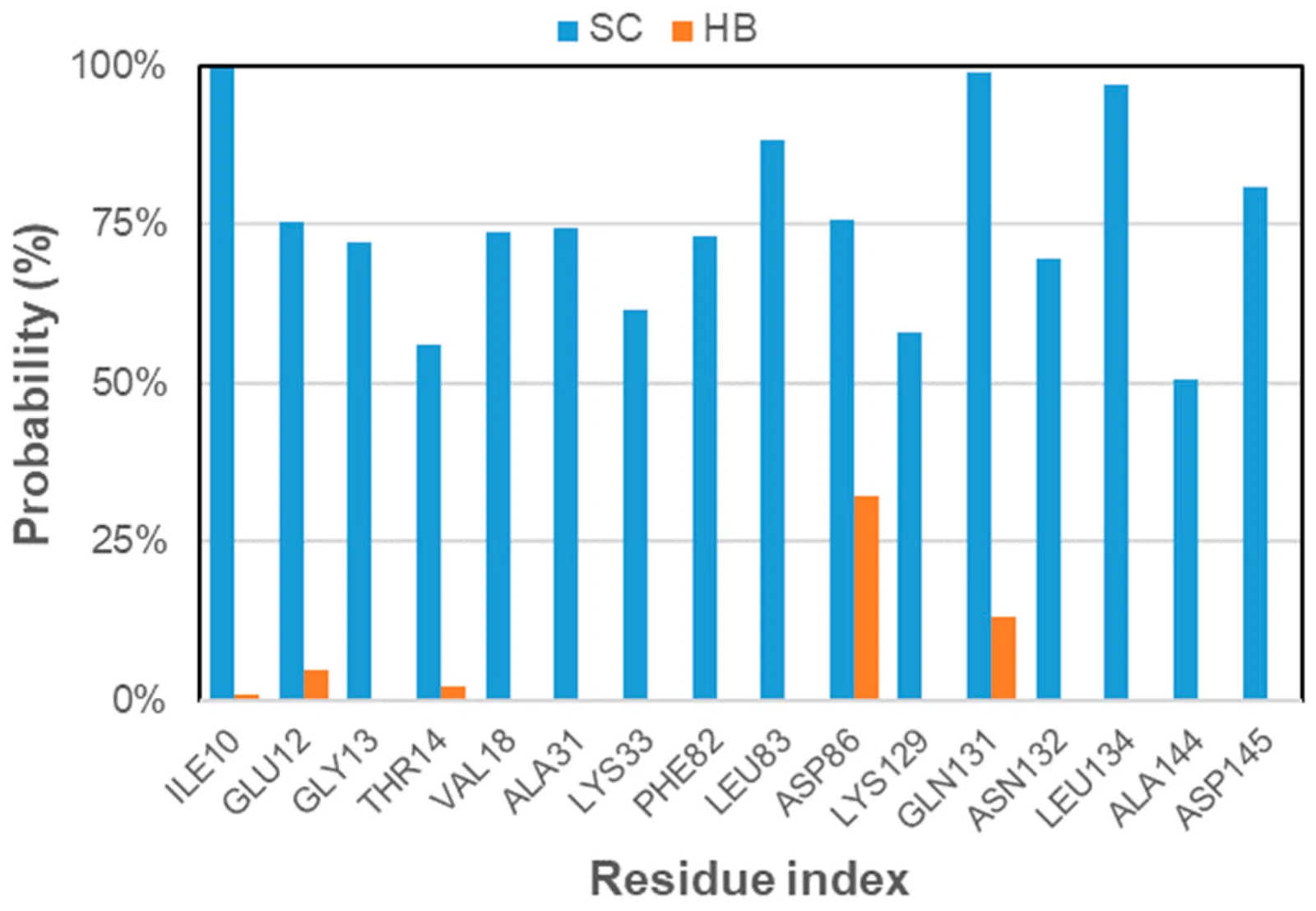
Key residues involved in forming SC and HB with the inhibitors. The obtained results were averaged over equilibrated snapshots of MD simulations.

Furthermore, examination of hydrogen-bonding (HB) interactions and side-chain (SC) contacts between CDK2 residues and ligands provides insight into the binding mechanisms of the inhibitors. Equilibrated MD snapshots of the CDK2-ligand complexes were analyzed to characterize intermolecular HB and SC interactions at the residue level. The residues involved in these contacts for each ligand are summarized in Table S7 of the Supporting Information. In particular, residues participating in both HB and SC interactions are highlighted in Figure 9.

These findings suggest that key residues—namely Ile10, Glu12, Gly13, Thr14, Val18, Ala31, Lys33, Phe82, Leu83, Asp86, Lys129, Gln131, Asn132, Leu134, Ala144, and Asp145—play important roles in governing ligand binding to CDK2.

### 2.7. Cell Viability Study

The three top-ranked compounds, **CID 636885**, **CID 46184320**, and **CID 101691758**, were identified from the ChemSpace library and ordered for cytotoxicity assays. The HGC-27 cell line (Human Stomach carcinoma cell) and HepG2 (Hepatocellular carcinoma) were selected as the target for evaluation of the cytotoxicity of the compounds using the dimethylthiazol-diphenyltetrazolium bromide (MTT) assay. The control cells showed high proliferation, which could be considered as 100%. Cells were treated with two potential compounds at various concentrations for 48 h.

The cytotoxic activity of the potential compounds, **CID 636885**, **CID 46184320**, and **CID 101691758**, were evaluated against HGC-27 and HepG2 cell lines (Table 5). All compounds demonstrated high proliferation inhibition against the HepG2 cell line, recording IC_50_ values of 65.79 ± 1.27 µg mL^−1^, 32.77 ± 0.56 µg mL^−1^ and 15.37 ± 0.46 µg mL^−1^, respectively. In contrast, the compounds exhibited moderate activity against the HGC-27 cell line, with IC_50_ values of 175.14 ± 2.78 µg mL^−1^ (**CID 636885**), 81.56 ± 1.72 µg mL^−1^ (**CID 46184320**) and 52.64 ± 1.33 µg mL^−1^ (**CID 101691758**).

## 3. Discussion

The present study employed an integrated computational workflow combining molecular docking, ADMET filtering, molecular dynamics simulations, and fast pulling of ligand calculations to identify alkaloid-derived compounds with potential CDK2-binding activity. The obtained results demonstrate that the combination of docking and nonequilibrium molecular dynamics approaches can effectively identify compounds with favorable binding affinity and interaction stability toward the ATP-binding pocket of CDK2.

The validation results obtained from the docking calculations revealed a moderate correlation between predicted and experimental binding free energies, yielding a Pearson correlation coefficient of R_Dock_ = 0.549 ± 0.180 together with an RMSE value of 4.68 ± 0.41 kcal mol^−1^. Although these values support the applicability of mVina as an initial virtual screening tool, the relatively large deviation from experimental data indicates that docking alone may not fully capture the complexity of protein–ligand interactions. This limitation is expected because molecular docking generally employs rigid receptor approximations, simplified scoring functions, and implicit treatment of solvent effects. Consequently, energetic contributions arising from conformational flexibility, solvent rearrangement, and long-range intermolecular interactions are only partially represented.

In contrast, the FPL simulations provided substantially improved predictive performance compared with docking calculations. At the selected pulling speed of k = 0.005 nm ps^−1^, the pulling work exhibited a correlation coefficient of R_W_ = −0.676 ± 0.119 with experimental binding free energies, accompanied by a significantly lower RMSE value of 2.22 ± 0.36 kcal mol^−1^. The improved Pearson correlation coefficient suggests that nonequilibrium molecular dynamics simulations provide a more realistic description of ligand dissociation energetics under near-physiological conditions. Since ligand unbinding requires disruption of multiple intermolecular interactions during the pulling process, the pulling work can better reflect the cumulative energetic contributions governing complex stability. Thus, it suggests that FPL simulations can serve as a computationally efficient alternative to more expensive free-energy perturbation approaches while maintaining reasonable predictive accuracy.

The strength of FPL approach, besides being beneficial for faster but still robust calculations, since the large-scale protein conformations at such short simulation times (700–7000 ps) remain generally in their original states, which is quite important. For kinases like CDK2, it might be active or inactive form, depending on if cyclin A or cyclin E is bound or not [6]. In addition, at the technical level, the Cα atoms are restrained during the pulling step also ensuring the large-scale motions do not happen. This setup prevents ligand binding estimations from being affected by significant, poorly controlled large-scale conformational reorganization of the protein target.

An important aspect of the study is the evaluation of different pulling speeds during FPL simulations. The obtained results indicate that lower pulling velocities generally improve the correlation between computed and experimental binding affinities. Specifically, the slowest pulling speed investigated (k = 0.0005 nm ps^−1^) yielded the highest correlation coefficients, with R_F_ = −0.680 and R_W_ = −0.681. However, the improvement compared with the faster pulling condition of k = 0.005 nm ps^−1^ (R_F_ = −0.652 and R_W_ = −0.676) was relatively small. In contrast, the computational cost increased substantially, with the simulation time increasing from approximately 700 ps to 7000 ps per trajectory. Therefore, the gain in predictive accuracy was not proportional to the additional computational expense. Considering both computational efficiency and predictive reliability, the pulling speed of k = 0.005 nm ps^−1^ was selected as the optimal condition for subsequent calculations. This parameter provides a practical balance between simulation throughput and predictive accuracy, which is particularly important for screening multiple ligand candidates in large-scale computational studies.

ADMET filtering was further implemented to improve the compound prioritization process by eliminating molecules with unfavorable mutagenicity or poor physicochemical properties. Among the 26 top-HIT compounds identified from docking simulations, **CID 636885**, **CID 46184320**, and **CID 101691758** were predicted to be non-mutagenic and exhibited acceptable lipophilicity values ranging from 1.69 to 4.97. In addition, these compounds maintained moderate aqueous solubility values between 113.4 and 155.5 mg L^−1^, indicating a suitable balance between hydrophobicity and solubility that may support favorable bioavailability characteristics.

Among the screened compounds, **CID 636885** exhibited the most favorable docking score of −19.4 kcal mol^−1^; whereas, **CID 46184320** and **CID 101691758** yielded less favorable scores of −17.5 and −17.4 kcal mol^−1^, respectively. However, subsequent MD and FPL analyses substantially refined this initial ranking. **CID 636885** remained predominantly stabilized by nonpolar contacts without forming persistent hydrogen bonds and exhibited the lowest pulling work and the least favorable FPL-derived binding free energy among the three candidates. Although hydrophobic contacts involving Ile10, Phe82, Gln131, and Leu134 contributed to ligand accommodation within the ATP-binding cavity, the absence of hydrogen bond interactions may reduce its long-term binding stability. In contrast, **CID 101691758** retained a key hydrogen bond with Asp86 and formed an additional hydrogen bond with Ile10 during MD simulations. It also produced the highest rupture force (795.6 ± 78.7 pN) and pulling work (89.2 ± 8.8 kcal mol^−1^), corresponding to the most favorable FPL-derived binding free energy ${∆G}_{FPL}^{Pre}$ = −6.48 kcal mol^−1^. **CID 46184320** exhibited a lower pulling work value of 80.9 ± 14.3 kcal mol^−1^ and a weaker predicted binding free energy of −5.78 kcal mol^−1^. Therefore, despite not achieving the best docking score, **CID 101691758** was identified as the most promising computational candidate. This re-ranking highlights the importance of MD/FPL refinement, which accounts for the dynamic stability and dissociation behavior of protein–ligand complexes that cannot be adequately captured by docking scores alone.

Despite the favorable MD/FPL results, the potential selectivity of these compounds toward CDK2 over other CDK family members cannot be established from the present study because the calculations were performed only against CDK2. This issue is particularly relevant because the ATP-binding sites of CDKs are highly conserved, which may result in cross-reactivity with closely related kinases. Comparative computational analyses against other CDKs may provide an initial indication of selectivity; however, biochemical profiling using a broader CDK panel is required for confirmation. Therefore, the identified compounds should currently be regarded as potential CDK2-binding candidates rather than selective CDK2 inhibitors.

Residue interaction analysis identified several amino acids, including Ile10, Glu12, Gly13, Thr14, Val18, Ala31, Lys33, Phe80, Phe82, Leu83, Asp86, Lys129, Gln131, Asn132, Leu134, Ala144, and Asp145, as key contributors to ligand recognition and stabilization. These residues collectively form a mixed hydrophobic-polar interaction network surrounding the ATP-binding cavity. In particular, residues such as Leu83 and Asp86 are known to play crucial roles in ATP-competitive inhibition of CDK2. The persistence of interactions involving these residues during MD simulations further supports the reliability of the obtained dock poses and suggests that the identified compounds adopt binding modes similar to previously reported type I CDK2 inhibitors.

Compared with previous computational studies on CDK2 inhibitors [15,16,17], the main distinction of the present work is not the use of docking or MD simulations themselves, but the implementation of a benchmarked FPL workflow for post-docking affinity ranking. While previous studies primarily relied on docking scores, MD stability, or end-point free-energy calculations for hit prioritization, the present study systematically optimized the FPL pulling speed using experimentally characterized CDK2 complexes before applying the protocol to a focused library of 2692 alkaloids. This strategy provided an additional layer of validation for ligand ranking and facilitated the identification of structurally distinct alkaloid-derived CDK2 inhibitor candidates.

It should be emphasized that the findings of this study are primarily based on computational approaches. While docking, MD, and FPL provide valuable insights into binding modes and stability, these predictions do not necessarily translate directly into biological activity. Therefore, preliminary experimental evaluation was performed using an MTT cell-viability assay against HepG2 and HGC-27 cancer cells. After 48 h of treatment, all three compounds reduced cell viability, with consistently lower IC_50_ values against HepG2 cells than against HGC-27 cells. The IC_50_ values of **CID 636885**, **CID 46184320**, and **CID 101691758** against HepG2 cells were 65.79 ± 1.27, 32.77 ± 0.56, and 15.37 ± 0.46 µg mL−^1^, respectively; whereas, the corresponding values against HGC-27 cells were 175.14 ± 2.78, 81.56 ± 1.72, and 52.64 ± 1.33 µg mL^−1^. Among the tested compounds, **CID 101691758** exhibited the highest cytotoxic effect, followed by **CID 46184320** and **CID 636885**. Nevertheless, the MTT assay measures an overall reduction in cell viability and does not demonstrate that the observed effects result from direct or selective CDK2 inhibition. Further biochemical CDK2 inhibition assays are required to establish the underlying mechanism of action.

## 4. Materials and Methods

### 4.1. Structure of Receptor and Ligand

Twenty experimentally characterized CDK2–ligand complexes were collected from the Protein Data Bank for validation of the docking and FPL protocols. Complexes were selected based on the availability of experimental CDK2 inhibitory data and a protein sequence identity exceeding 97% relative to the reference CDK2 sequence (UniProt P24941) (https://www.uniprot.org/uniprotkb/P24941/entry, accessed on 9 January 2026). The benchmark set comprised the following PDB entries: 1V1K [27], 2I40 [28], 2R3H [29], 2R3I [29], 2R3J [29], 2VV9 [30], 2W05 [31], 2W17 [32], 3DDQ [33], 3EID [34], 3LFS [35], 3MY5 [36], 3NS9 [37], 3PJ8 [38], 3PXY [39], 3PY0 [39], 3S2P [40], 4CFX [41], 4RJ3 [42], and 6GVA [43]. The benchmark set was constructed based on the availability of experimentally characterized CDK2–ligand complexes and corresponding inhibitory data, rather than the primary therapeutic target investigated in the original studies. Accordingly, PDB ID 4RJ3 was retained because it provides a validated CDK2–ligand complex with reported CDK2 inhibitory activity, although the associated publication primarily focused on EGFR inhibitor development.

A structure-based alkaloid library was assembled from PubChem by querying the Medical Subject Headings (MeSH) descriptor “Alkaloids.” Of the 3273 retrieved entries, compounds lacking an available three-dimensional conformer or failing successful ligand preparation in AutoDockTools 1.5.6rc3 were excluded. The resulting screening library comprised 2692 alkaloids.

### 4.2. Molecular Docking Simulations

A modified version of AutoDock Vina (mVina) [18], incorporating adjusted empirical parameters to enhance ligand ranking, was applied to dock the inhibitors into the CDK2 active site. The top-ranked poses were selected based on the most favorable binding energies. Parameterization of proteins and ligands was performed by AutoDockTools, adding hydrogens and partial charges. The docking grid size was set to 24 × 24 × 24 Å, ensuring coverage of the active site. Docking was performed using the default exhaustiveness value of 8, and the maximum energy difference between predicted binding modes was set to 7 kcal mol^−1^, also following the default setting.

### 4.3. Molecular Dynamics Simulations

MD simulations were carried out to confirm the findings from the docking analysis. All calculations were performed with GROMACS 2019.6, while the protein and ionic components were described using the Amber99SB-ILDN force field [44]. Water molecules were parameterized using the TIP3P water model [45]. The combination of the AMBER99SB-ILDN force field with the TIP3P solvent model has been recognized as an effective approach for reliable free energy estimation [46]. Ligand parameterization was subsequently performed using the General AMBER Force Field (GAFF), with topologies and parameters generated via the AmberTools18 suite and the ACPYPE packages [47]. The properties of the alkaloid compounds were determined through quantum chemical calculations employing the MP2 level with the 6–31G(d,p) basis set and an implicit solvent model (ε = 78.4). All computations were carried out using Gaussian 09. Notably, atomic partial charges were assigned according to the restrained electrostatic potential (RESP) method [48]. Each CDK2-ligand complex was embedded in a dodecahedral simulation box with an approximate volume of 573 nm^3^, containing nearly 56,000 atoms in total.

Following parameterization, the system underwent energy minimization using the steepest descent algorithm. A time step of 2 fs was applied for all MD simulations, which were performed at 300 K and 1 atm using an appropriate integrator. A cutoff distance of 0.9 nm was used for non-bonded interactions. System equilibration was performed in two sequential steps, consisting of a 100 ps NVT equilibration followed by a 100 ps NPT equilibration. During both stages, positional restraints were imposed on the C_α_ atoms, with a force constant of 1000 kJ mol^−1^ nm^−2^ applied to the heavy atoms of the protein–ligand complex. The final configuration from the NPT phase served as the starting point for the production simulation, which was carried out for 100 ns. To improve sampling reliability and reproducibility, the full simulation protocol was independently repeated four times.

### 4.4. Steered-Molecular Dynamics Simulations

**Figure 10:**
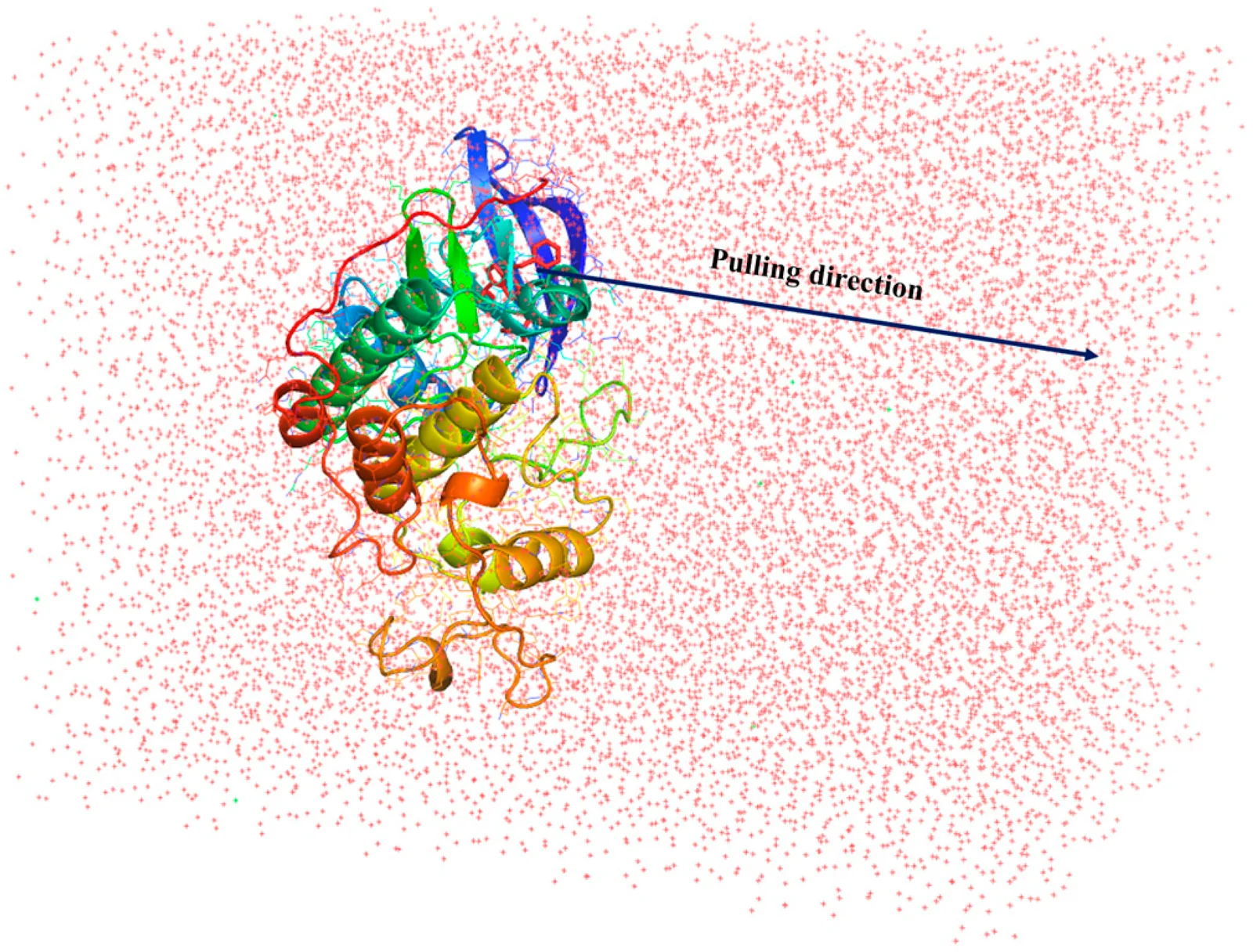
Initial conformation of FPL simulations of the CDK2 + inhibitor.

For each CDK2–ligand complex, an equilibrated configuration extracted from the stable portion of the 100 ns production MD trajectory was used as the initial structure for the subsequent steered molecular dynamics simulations. In these simulations, the alkaloid ligands were mobilized out of the CDK2 binding pocket. The complex of CDK2 and inhibitor was covered into a rectangular periodic boundary conditions box with a size of 7.8 × 7.6 × 9.8 nm which is large enough for unbound ligands out of the protein binding cavity. More than 50,000 atoms including CDK2 protein, inhibitor, water molecules and counter-balanced ions Cl^−^, which are equivalent to 579 nm^3^ of volume, were involved in the simulated system. During the process, the CDK2 C_α_ atoms were positionally fixed by a weak harmonic force of 1000 kJ mol^−1^ nm^−2^ in three dimensions. The inhibitor was dissociated via an external harmonic force with a cantilever of a spring constant of 600 kJ mol^−1^ nm^−2^. To evaluate the impact of pulling speeds, the dissociation velocity was set at various constant values, specifically 0.005, 0.002, 0.001, and 0.0005 nm ps^−1^. The external harmonic force was applied on the center of mass of the CDK2 inhibitor along the Z direction (Figure 10). In FPL simulations, ligand-binding affinity is associated with the magnitude of the pulling work, which is connected to the binding free energy according to the Jarzynski equality. During the simulation process, the pulling force and ligand displacement were monitored every 10 ps to assess the interaction strength between the ligand and CDK2. To improve conformational sampling, 4 independent non-equilibrium molecular dynamics trajectories were generated for each ligand–CDK2 complex using identical initial structures but different randomly assigned initial velocities. The pulling work was subsequently determined as the mean value obtained from the four FPL simulations.

### 4.5. Analysis Tools

The protonation states of the ligands were determined using the Chemicalize web platform provided by ChemAxon (http://www.chemicalize.com). A hydrogen bond was considered present when the acceptor–hydrogen-donor angle exceeded 135° and the acceptor–donor distance was below 0.35 nm. Side-chain interactions were identified when any non-hydrogen atom of CDK2 was within 0.45 nm of a ligand atom. Computational uncertainties were estimated by performing 20,000 bootstrap resampling iterations. Three-dimensional protein–ligand interaction diagrams were generated using the PLIP web server, and structural visualizations were prepared using PyMOL 3.1.3. Additionally, the PreADMET web server was used to evaluate key metrics related to the compounds’ ability [49].

### 4.6. Cell Lines and Cell Culture

The HGC-27 (Human Stomach carcinoma cell) and HepG2 (Hepatocellular carcinoma) cell lines were obtained from ATCC (Manassas, VA, USA) and maintained at 37 °C in 5% CO_2_ in suitable media (RPMI 1640, MEM, DMEM; Merck KGaA, Darmstadt, Germany) containing 10% heat-inactivated fetal bovine serum (FBS), penicillin (100 UI mL^−1^), streptomycin (100 mg mL^−1^), and L-glutamine (2 mM).

### 4.7. Cytotoxic Assay

Cell viability was assessed through MTT [3-(4,5-dimethylthiazol-2-yl)-2,5-diphenyltetrazolium bromide] assay [50,51] which was adopted to measure the in vitro cytotoxicity of the nanoparticles in the cancer cell lines. Briefly, the cells were seeded in a 96-well plate (5 × 10^4^ cells per well of 200 µL mixture) and attached for 24 h. The cells were subsequently treated with two HIT compounds in the range 100 to 1 μg.mL^−1^ and incubated at 37 °C and 5% CO_2_ for 48 h. The control was selected as ellipticine. At the end of incubations, 20 μL of MTT (Merck KGaA) was added to the wells and incubated at 37 °C for 4 h. Absorbance was recorded at 540/720 nm by using a Spark multimode reader (Tecan, Männedorf, Switzerland). The growth inhibition was assessed using the following formula: Inhibition rate (%) = (1−OD_sample_/OD_con_) ×100%, where OD_sample_ and OD_con_ are the optical densities of the experimental sample groups and control, respectively. The aforementioned experiment was carried out three times to get average IC_50_ values using TableCurve AISN Software (Jandel Scientific, San Rafael, CA, USA). Cytotoxicity is expressed as an IC_50_ value (the concentration of test agents that cause 50% inhibition or cell death), which was obtained by nonlinear regression using the TableCurve 2D program (SPSS Statistical Software, Chicago, IL, USA).

## 5. Conclusions

In the present study, the docking and FPL protocols were validated using 20 experimentally characterized inhibitors of CDK2. The binding free energies of the CDK2-ligand complexes were estimated from experimental IC_50_ value (ΔG_exp_), molecular docking (ΔG_Dock_), and FPL simulations (${\Delta G}_{FPL}^{Pre}$). A strong correlation was observed between ΔG_exp_ and ${\Delta G}_{FPL}^{Pre}$ (R_W_ = −0.676 ± 0.119 and RMSE_W_ = 2.22 ± 0.36 kcal mol^−1^). In contrast, the dock scores showed only moderate agreement with the experimental data, yielding an R_Dock_ = 0.549 ± 0.180 and RMSE = 4.68 ± 0.41 kcal mol^−1^. These findings suggest that the FPL approach provides superior predictive performance for evaluating CDK2 ligand-binding affinity. Following virtual screening of 2692 alkaloid compounds against the CDK2 binding pocket, 26 high-ranked compounds were selected for further investigation. The obtained results were then filtered for ADMET properties and refined *via* FPL simulations. Three compounds (**CID 636885**, **CID 46184320** and **CID 101691758**) were identified as candidates with favorable predicted binding to the CDK2 ATP-binding pocket because they have low ${\Delta G}_{FPL}^{Pre}$ which varies from −5.17 to −6.48 kcal mol^−1^. In addition, the residues including Ile10, Glu12, Gly13, Thr14, Val18, Ala31, Lys33, Phe80, Phe82, Leu83, Asp86, Lys129, Gln131, Asn132, Leu134, Ala144 and Asp145 play a crucial role in the CDK2 binding process. Our preliminary cytotoxicity assay showed all three candidates as effective anti-proliferative molecules against the HepG2 cell line. Further biochemical evaluation, including comparative kinase-panel assays, is required to confirm both the CDK2 inhibitory activity and selectivity of the identified compounds over other CDK family members.

## Figures and Tables

**Table 1:** Binding free energies (kcal mol^−1^) for experimentally validated inhibitors of CDK2 obtained by docking simulation.

No.	PDB ID	Sequence Similarity (%)	IC_50_ (µM)	ΔG_exp_ (kcal mol^−1^)	ΔG_Dock_ (kcal mol^−1^)
1	2W05	100	0.001	−12.76	−14.50
2	2W17	100	0.002	−12.34	−15.90
3	3NS9	100	0.003	−12.09	−14.80
4	6GVA	99.67	0.006	−11.66	−14.70
5	2R3J	100	0.010	−11.35	−13.30
6	2VV9	100	0.017	−11.02	−15.20
7	3PJ8	99.67	0.040	−10.49	−13.70
8	3S2P	100	0.068	−10.17	−12.80
9	4RJ3	100	0.093	−9.97	−14.80
10	3PY0	97.39	0.130	−9.77	−12.20
11	3DDQ	99.67	0.210	−9.47	−14.30
12	3EID	100	0.450	−9.00	−15.20
13	2R3I	100	1.000	−8.51	−13.60
14	2I40	100	1.580	−8.23	−15.40
15	3LFS	100	2.500	−7.95	−12.70
16	3PXY	97.39	5.900	−7.42	−12.10
17	4CFX	98.35	8.700	−7.18	−13.70
18	2R3H	100	20.000	−6.66	−10.60
19	1V1K	100	35.000	−6.32	−14.80
20	3MY5	99.33	65	−5.94	−10.80

**Table 2:** Twenty-six highest-ranked compounds predicted by mVina results and their ADMET properties.

No.	CID ID	Dock Score (kcal mol^−1^)	Ro5 Violations	alogP	Water Solubility (mg L^−1^)	Ames Test
1	636885	−19.4	1	1.69	134.5	non-mutagen
2	193239	−19.1	2	5.38	313.5	non-mutagen
3	101168510	−18.6	3	−0.37	903.0	mutagen
4	132776	−18.5	2	5.02	66.6	non-mutagen
5	122181663	−18.2	2	4.47	26.5	non-mutagen
6	44576164	−18.2	1	4.98	32.2	mutagen
7	188390	−18.1	2	5.53	647.5	mutagen
8	440585	−18.1	2	5.16	312.0	non-mutagen
9	51106	−18.1	2	5.76	136.2	non-mutagen
10	10031670	−17.8	2	5.77	130.7	non-mutagen
11	129021	−17.8	2	4.42	312.9	non-mutagen
12	44559871	−17.8	1	5.72	500.1	mutagen
13	46184320	−17.5	1	4.32	155.5	non-mutagen
14	5280954	−17.5	1	4.48	1.89	mutagen
15	5320557	−17.5	0	4.93	40.1	mutagen
16	101691758	−17.4	1	4.97	113.4	non-mutagen
17	25158703	−17.4	1	3.92	256.1	mutagen
18	73400	−17.4	2	5.97	70.0	non-mutagen
19	44206241	−17.3	1	3.64	742.0	mutagen
20	6769	−17.3	2	4.39	6.05	non-mutagen
21	107715	−17.2	2	3.56	697.50	non-mutagen
22	10865014	−17.1	1	6.22	5.71	mutagen
23	157828	−17.1	1	5.34	298.30	mutagen
24	5471853	−17.1	2	4.92	53.62	non-mutagen
25	133012	−17.0	2	3.39	418.60	non-mutagen
26	54803	−17.0	1	3.49	414.60	mutagen

**Table 3:** FPL results of pulling work (W) in 20 experimental complexes.

No.	PDB ID	ΔG_exp_ (kcal mol^−1^)	W (kcal mol^−1^)			
			k = 0.005	k = 0.002	k = 0.001	k = 0.0005
1	2W05	−12.76	163.9 ± 15.6	122.3 ± 8.9	110.1 ± 8.0	78.3 ± 12.0
2	2W17	−12.34	203.1 ± 10.6	166.3 ± 14.4	142.7 ± 15.4	103.5 ± 4.3
3	3NS9	−12.09	207.7 ± 7.6	144.2 ± 13.1	138.3 ± 9.7	116.5 ± 5.2
4	6GVA	−11.66	119.4 ± 10.9	101.0 ± 11.7	76.6 ± 1.1	71.8 ± 2.3
5	2R3J	−11.35	112.7 ± 8.4	93.1 ± 9.5	79.6 ± 7.2	71.7 ± 8.1
6	2VV9	−11.02	132.7 ± 10.5	99.5 ± 4.9	81.3 ± 10.3	84.5 ± 6.6
7	3PJ8	−10.49	162.5 ± 11.7	133.6 ± 6.4	115.5 ± 9.0	98.0 ± 7.6
8	3S2P	−10.17	126.4 ± 9.3	62.7 ± 3.1	56.7 ± 5.7	47.1 ± 5.7
9	4RJ3	−9.97	68.3 ± 5.2	64.8 ± 4.3	42.6 ± 3.3	45.6 ± 3.6
10	3PY0	−9.77	130.2 ± 4.9	84.8 ± 3.1	89.0 ± 3.6	88.4 ± 9.2
11	3DDQ	−9.47	112.4 ± 7.6	95.8 ± 7.4	74.7 ± 5.9	64.6 ± 8.5
12	3EID	−9.00	93.9 ± 5.9	81.7 ± 6.0	64.3 ± 3.6	56.4 ± 3.3
13	2R3I	−8.51	106.8 ± 11.0	95.9 ± 5.5	86.7 ± 3.6	66.8 ± 4.2
14	2I40	−8.23	125.0 ± 9.6	104.0 ± 13.3	78.7 ± 5.7	60.4 ± 0.3
15	3LFS	−7.95	126.7 ± 13.2	131.8 ± 17.9	99.6 ± 6.3	79.9 ± 7.7
16	3PXY	−7.42	101.0 ± 5.3	80.2 ± 3.3	65.0 ± 2.4	51.4 ± 2.0
17	4CFX	−7.18	117.1 ± 10.9	75.5 ± 6.1	68.8 ± 6.0	67.7 ± 3.0
18	2R3H	−6.66	77.2 ± 7.2	58.8 ± 2.0	49.4 ± 1.9	47.6 ± 4.2
19	1V1K	−6.32	107.8 ± 11.6	77.3 ± 6.7	69.0 ± 1.9	48.4 ± 2.8
20	3MY5	−5.94	83.3 ± 4.9	62.8 ± 3.6	40.5 ± 1.2	37.4 ± 3.2

**Table 4:** The computational values of three prioritized compounds using molecular docking and FPL simulations.

No.	CID ID	ΔG_dock_ (kcal mol^−1^)	${∆G}_{FPL}^{Pre}$ (kcal mol^−1^)	F_max_ (pN)	W (kcal mol^−1^)	${IC}_{50}^{Pre}$ (µM)
1	636885	−19.4	−5.17	654.1 ± 104.9	73.6 ± 11.7	~224.0
2	46184320	−17.5	−5.78	682.5 ± 80.0	80.9 ± 14.3	~82.8
3	101691758	−17.4	−6.48	795.6 ± 78.7	89.2 ± 8.8	~26.7

**Table 5:** Cytotoxic activity (IC_50_, µg mL^−1^) of potential compounds on HGC-27 and HepG2 cell lines after 48 h of incubation.

No.	PubChem ID	HGC-27 ^a^	HepG2 ^a^
1	CID 636885	175.14 ± 2.78	65.79 ± 1.27
2	CID 46184320	81.56 ± 1.72	32.77 ± 0.56
3	CID 101691758	52.64 ± 1.33	15.37 ± 0.46
4	ellipticine	0.48 ± 0.17	0.52 ± 0.11

^a^ IC_50_ value (µM mL^−1^). Results are expressed as the mean ± SD values from three independent experiments.

## Data Availability

The original contributions presented in this study are included in the article. Further inquiries can be directed to the corresponding authors.

## Supplementary Materials

The following supporting information can be downloaded at: https://www.mdpi.com/article/10.3390/ph19081320/s1. Table S1. Graph of Fmax and W of twenty experimental complexes over time (pulling speed of k = 0.005 nm ps^−1^). Table S2. Graph of Fmax and W of twenty experimental complexes over time (pulling speed of k = 0.002 nm ps^−1^). Table S3. Graph of Fmax and W of twenty experimental complexes over time (pulling speed of k = 0.001 nm ps^−1^). Table S4. Graph of Fmax and W of twenty experimental complexes over time (pulling speed of k = 0.0005 nm ps^−1^). Table S5. FPL results of maximum pulling force (Fmax) of 20 experimental structures. Table S6. Graph of Fmax and W of three top-lead compounds over time (pulling speed of k = 0.005 nm ps^−1^). Table S7. List of CDK2 residues forming SC/HB contacts to the three top-lead compounds. Figure S1. Correlation of Fmax and ΔGexp values (pulling speed of k = 0.005 nm ps^−1^). Figure S2. Correlation of Fmax and ΔGexp values (pulling speed of k = 0.002 nm ps^−1^). Figure S3. Correlation of Fmax and ΔGexp values (pulling speed of k = 0.001 nm ps^−1^). Figure S4.Correlation of Fmax and ΔGexp values (pulling speed of k = 0.0005 nm ps^−1^). Figure S5. Correlation of W and ΔGexp values (pulling speed of k = 0.005 nm ps^−1^). Figure S6. Correlation of W and ΔGexp values (pulling speed of k = 0.002 nm ps^−1^). Figure S7. Correlation of W and ΔGexp values (pulling speed of k = 0.001 nm ps^−1^). Figure S8. Correlation of W and ΔGexp values (pulling speed of k = 0.0005 nm ps^−1^).

## References

[1] Hanahan D., Weinberg R.A. (2011). Hallmarks of Cancer: The Next Generation. Cell.

[2] Sung H., Ferlay J., Siegel R.L., Laversanne M., Soerjomataram I., Jemal A., Bray F. (2021). Global Cancer Statistics 2020: GLOBOCAN Estimates of Incidence and Mortality Worldwide for 36 Cancers in 185 Countries. CA Cancer J. Clin..

[3] Malumbres M., Barbacid M. (2009). Cell cycle, CDKs and cancer: A changing paradigm. Nat. Rev. Cancer.

[4] Asghar U., Witkiewicz A.K., Turner N.C., Knudsen E.S. (2015). The history and future of targeting cyclin-dependent kinases in cancer therapy. Nat. Rev. Drug Discov..

[5] Spring L.M., Wander S.A., Zangardi M., Bardia A. (2019). CDK 4/6 Inhibitors in Breast Cancer: Current Controversies and Future Directions. Curr. Oncol. Rep..

[6] Tadesse S., Caldon E.C., Tilley W., Wang S. (2018). Cyclin-Dependent Kinase 2 Inhibitors in Cancer Therapy: An Update. J. Med. Chem..

[7] Dietrich C., Trub A., Ahn A., Taylor M., Ambani K., Chan K.T., Lu K.-H., Mahendra C.A., Blyth C., Coulson R. (2024). INX-315, a Selective CDK2 Inhibitor, Induces Cell Cycle Arrest and Senescence in Solid Tumors. Cancer Discov..

[8] Dommer A.P., Kumarasamy V., Wang J., O’Connor T.N., Roti M., Mahan S., McLean K., Knudsen E.S., Witkiewicz A.K. (2025). Tumor Suppressors Condition Differential Responses to the Selective CDK2 Inhibitor BLU-222. Cancer Res..

[9] Newman D.J., Cragg G.M. (2020). Natural Products as Sources of New Drugs over the Nearly Four Decades from 01/1981 to 09/2019. J. Nat. Prod..

[10] Li Q., Shah S. (2017). Structure-Based Virtual Screening. Protein Bioinformatics.

[11] Abramson J., Adler J., Dunger J., Evans R., Green T., Pritzel A., Ronneberger O., Willmore L., Ballard A.J., Bambrick J. (2024). Accurate structure prediction of biomolecular interactions with AlphaFold 3. Nature.

[12] Xu Z., Ren F., Wang P., Cao J., Tan C., Ma D., Zhao L., Dai J., Ding Y., Fang H. (2025). A generative AI-discovered TNIK inhibitor for idiopathic pulmonary fibrosis: A randomized phase 2a trial. Nat. Med..

[13] Trott O., Olson A.J. (2010). AutoDock Vina: Improving the speed and accuracy of docking with a new scoring function, efficient optimization, and multithreading. J. Comput. Chem..

[14] Singh N., Li W. (2020). Absolute Binding Free Energy Calculations for Highly Flexible Protein MDM2 and Its Inhibitors. Int. J. Mol. Sci..

[15] Lu F., Luo G., Qiao L., Jiang L., Li G., Zhang Y. (2016). Virtual Screening for Potential Allosteric Inhibitors of Cyclin-Dependent Kinase 2 from Traditional Chinese Medicine. Molecules.

[16] Mahajan P., Chashoo G., Gupta M., Kumar A., Singh P.P., Nargotra A. (2017). Fusion of Structure and Ligand Based Methods for Identification of Novel CDK2 Inhibitors. J. Chem. Inf. Model..

[17] Chagaleti B.K., Kumar S., Anjana G.V., Rajagopal R., Alfarhan A., Arockiaraj J., Muthu Kumaradoss K., Karthick Raja Namasivayam S. (2024). Targeting cyclin-dependent kinase 2 CDK2: Insights from molecular docking and dynamics simulation—A systematic computational approach to discover novel cancer therapeutics. Comput. Biol. Chem..

[18] Pham T.N.H., Nguyen T.H., Tam N.M., Vu T.Y., Pham N.T., Huy N.T., Mai B.K., Tung N.T., Pham M.Q., Vu V.V. (2021). Improving ligand-ranking of AutoDock Vina by changing the empirical parameters. J. Comput. Chem..

[19] Ngo S.T., Tam N.M., Pham M.Q., Nguyen T.H. (2021). Benchmark of Popular Free Energy Approaches Revealing the Inhibitors Binding to SARS-CoV-2 Mpro. J. Chem. Inf. Model..

[20] Nguyen T.H., Tran P.-T., Pham N.Q.A., Hoang V.-H., Hiep D.M., Ngo S.T. (2022). Identifying Possible AChE Inhibitors from Drug-like Molecules via Machine Learning and Experimental Studies. ACS Omega.

[21] Caffalette C.A., Corey R.A., Sansom M.S.P., Stansfeld P.J., Zimmer J. (2019). A lipid gating mechanism for the channel-forming O antigen ABC transporter. Nat. Commun..

[22] Grither W.R., Longmore G.D. (2018). Inhibition of tumor–microenvironment interaction and tumor invasion by small-molecule allosteric inhibitor of DDR2 extracellular domain. Proc. Natl. Acad. Sci. USA.

[23] Duca J.S., Madison V.S., Voigt J.H. (2008). Cross-Docking of Inhibitors into CDK2 Structures. 1. J. Chem. Inf. Model..

[24] Gowthaman U., Jayakanthan M., Sundar D. (2008). Molecular docking studies of dithionitrobenzoic acid and its related compounds to protein disulfide isomerase: Computational screening of inhibitors to HIV-1 entry. BMC Bioinform..

[25] Ngo S.T., Nguyen T.H., Tung N.T., Vu V.V., Pham M.Q., Mai B.K. (2022). Characterizing the ligand-binding affinity toward SARS-CoV-2 Mproviaphysics- and knowledge-based approaches. Phys. Chem. Chem. Phys..

[26] Ngo S.T., Nguyen M.T., Nguyen M.T. (2017). Determination of the absolute binding free energies of HIV-1 protease inhibitors using non-equilibrium molecular dynamics simulations. Chem. Phys. Lett..

[27] Beattie J.F., Breault G.A., Ellston R.P.A., Green S., Jewsbury P.J., Midgley C.J., Naven R.T., Minshull C.A., Pauptit R.A., Tucker J.A. (2003). Cyclin-dependent kinase 4 inhibitors as a treatment for cancer. Part 1: Identification and optimisation of substituted 4,6-Bis anilino pyrimidines. Bioorg. Med. Chem. Lett..

[28] Bamborough P., Christopher J.A., Cutler G.J., Dickson M.C., Mellor G.W., Morey J.V., Patel C.B., Shewchuk L.M. (2006). 5-(1H-Benzimidazol-1-yl)-3-alkoxy-2-thiophenecarbonitriles as potent, selective, inhibitors of IKK-ε kinase. Bioorg. Med. Chem. Lett..

[29] Fischmann T.O., Hruza A., Duca J.S., Ramanathan L., Mayhood T., Windsor W.T., Le H.V., Guzi T.J., Dwyer M.P., Paruch K. (2007). Structure-guided discovery of cyclin-dependent kinase inhibitors. Biopolymers.

[30] Finlay M.R.V., Acton D.G., Andrews D.M., Barker A.J., Dennis M., Fisher E., Graham M.A., Green C.P., Heaton D.W., Karoutchi G. (2008). Imidazole piperazines: SAR and development of a potent class of cyclin-dependent kinase inhibitors with a novel binding mode. Bioorg. Med. Chem. Lett..

[31] Anderson M., Andrews D.M., Barker A.J., Brassington C.A., Breed J., Byth K.F., Culshaw J.D., Finlay M.R.V., Fisher E., McMiken H.H.J. (2008). Imidazoles: SAR and development of a potent class of cyclin-dependent kinase inhibitors. Bioorg. Med. Chem. Lett..

[32] Jones C.D., Andrews D.M., Barker A.J., Blades K., Daunt P., East S., Geh C., Graham M.A., Johnson K.M., Loddick S.A. (2008). The discovery of AZD5597, a potent imidazole pyrimidine amide CDK inhibitor suitable for intravenous dosing. Bioorg. Med. Chem. Lett..

[33] Bettayeb K., Oumata N., Echalier A., Ferandin Y., Endicott J.A., Galons H., Meijer L. (2008). CR8, a potent and selective, roscovitine-derived inhibitor of cyclin-dependent kinases. Oncogene.

[34] Stevens K.L., Reno M.J., Alberti J.B., Price D.J., Kane-Carson L.S., Knick V.B., Shewchuk L.M., Hassell A.M., Veal J.M., Davis S.T. (2008). Synthesis and evaluation of pyrazolo [1,5-b] pyridazines as selective cyclin dependent kinase inhibitors. Bioorg. Med. Chem. Lett..

[35] Lesuisse D., Dutruc-Rosset G., Tiraboschi G., Dreyer M.K., Maignan S., Chevalier A., Halley F., Bertrand P., Burgevin M.-C., Quarteronet D. (2010). Rational design of potent GSK3β inhibitors with selectivity for Cdk1 and Cdk2. Bioorg. Med. Chem. Lett..

[36] Baumli S., Endicott J.A., Johnson L.N. (2010). Halogen Bonds Form the Basis for Selective P-TEFb Inhibition by DRB. Chem. Biol..

[37] Heathcote D.A., Patel H., Kroll S.H.B., Hazel P., Periyasamy M., Alikian M., Kanneganti S.K., Jogalekar A.S., Scheiper B., Barbazanges M. (2010). A Novel Pyrazolo [1,5-a] pyrimidine Is a Potent Inhibitor of Cyclin-Dependent Protein Kinases 1, 2, and 9, Which Demonstrates Antitumor Effects in Human Tumor Xenografts Following Oral Administration. J. Med. Chem..

[38] Jorda R., Havlíček L., McNae I.W., Walkinshaw M.D., Voller J., Šturc A., Navrátilová J., Kuzma M., Mistrík M., Bártek J. (2011). Pyrazolo [4,3-d] pyrimidine Bioisostere of Roscovitine: Evaluation of a Novel Selective Inhibitor of Cyclin-Dependent Kinases with Antiproliferative Activity. J. Med. Chem..

[39] Betzi S., Alam R., Martin M., Lubbers D.J., Han H., Jakkaraj S.R., Georg G.I., Schönbrunn E. (2011). Discovery of a Potential Allosteric Ligand Binding Site in CDK2. ACS Chem. Biol..

[40] Lee J., Kim K.-H., Jeong S. (2011). Discovery of a novel class of 2-aminopyrimidines as CDK1 and CDK2 inhibitors. Bioorg. Med. Chem. Lett..

[41] Carbain B., Paterson D.J., Anscombe E., Campbell A.J., Cano C., Echalier A., Endicott J.A., Golding B.T., Haggerty K., Hardcastle I.R. (2013). 8-Substituted O6-Cyclohexylmethylguanine CDK2 Inhibitors: Using Structure-Based Inhibitor Design to Optimize an Alternative Binding Mode. J. Med. Chem..

[42] Hanan E.J., Eigenbrot C., Bryan M.C., Burdick D.J., Chan B.K., Chen Y., Dotson J., Heald R.A., Jackson P.S., La H. (2014). Discovery of Selective and Noncovalent Diaminopyrimidine-Based Inhibitors of Epidermal Growth Factor Receptor Containing the T790M Resistance Mutation. J. Med. Chem..

[43] Jorda R., Havlíček L., Šturc A., Tušková D., Daumová L., Alam M., Škerlová J., Nekardová M., Peřina M., Pospíšil T. (2019). 3,5,7-Substituted Pyrazolo [4,3-d] pyrimidine Inhibitors of Cyclin-Dependent Kinases and Their Evaluation in Lymphoma Models. J. Med. Chem..

[44] Aliev A.E., Kulke M., Khaneja H.S., Chudasama V., Sheppard T.D., Lanigan R.M. (2014). Motional timescale predictions by molecular dynamics simulations: Case study using proline and hydroxyproline sidechain dynamics. Proteins Struct. Funct. Bioinform..

[45] Jorgensen W.L., Chandrasekhar J., Madura J.D., Impey R.W., Klein M.L. (1983). Comparison of simple potential functions for simulating liquid water. J. Chem. Phys..

[46] Zhang H., Yin C., Jiang Y., van der Spoel D. (2018). Force Field Benchmark of Amino Acids: I. Hydration and Diffusion in Different Water Models. J. Chem. Inf. Model..

[47] Sousa da Silva A.W., Vranken W.F. (2012). ACPYPE-AnteChamber PYthon Parser interface. BMC Res. Notes.

[48] Wang J., Wolf R.M., Caldwell J.W., Kollman P.A., Case D.A. (2004). Development and testing of a general amber force field. J. Comput. Chem..

[49] Lee S.K., Lee I.H., Kim H.J., Chang G.S., Chung J.E., No K.T. (2003). The PreADME Approach: Web-based program for rapid prediction of physico-chemical, drug absorption and drug-like properties. EuroQSAR 2002 EuroQSAR 2002 Designing Drugs and Crop Protectants: Processes, Problems and Solutions.

[50] Mosmann T. (1983). Rapid colorimetric assay for cellular growth and survival: Application to proliferation and cytotoxicity assays. J. Immunol. Methods.

[51] Yang L., Tu D., Zhao Z., Cui J. (2017). Cytotoxicity and apoptosis induced by mixed mycotoxins (T-2 and HT-2 toxin) on primary hepatocytes of broilers in vitro. Toxicon.

