# Supplementary material for: An Integrated Computational Workflow for Discovering Alkaloid-Derived Ligands of Cyclin-Dependent Kinase 2
Source: Pharmaceuticals (Basel). 2026 Aug 21;19(8):1320. doi: 10.3390/ph19081320 (PMC13516633; doi:10.3390/ph19081320)
Supplement: Supplementary file 1 [file pharmaceuticals-19-01320-s001.zip › pharmaceuticals-4429154-supplementary.pdf]

An integrated computational workflow for discovering alka-loid-derived ligands of cyclin-dependent kinase 2

Content

Table S1. Graph of F_{max} and W of twenty experimental complexes over time (pulling speed of k = 0.005 nm ps^{-1})........2
Table S2. Graph of F_{max} and W of twenty experimental complexes over time (pulling speed of k = 0.002 nm ps^{-1})........6
Table S3. Graph of F_{max} and W of twenty experimental complexes over time (pulling speed of k = 0.001 nm ps^{-1})........11
Table S4. Graph of F_{max} and W of twenty experimental complexes over time (pulling speed of k = 0.0005 nm ps^{-1})........16
Table S5. FPL results of maximum pulling force (F_{max}) of 20 experimental structures 21
Table S6. Graph of F_{max} and W of three top-lead compounds over time (pulling speed of k = 0.005 nm ps^{-1})........22
Table S7. List of CDK2 residues forming SC/HB contacts to the three top-lead compounds......23
Figure S1. Correlation of F_{max} and ΔG_{exp} values (pulling speed of k = 0.005 nm ps^{-1}) 25
Figure S2. Correlation of F_{max} and ΔG_{exp} values (pulling speed of k = 0.002 nm ps^{-1}) 25
Figure S3. Correlation of F_{max} and ΔG_{exp} values (pulling speed of k = 0.001 nm ps^{-1}) 25
Figure S4. Correlation of F_{max} and ΔG_{exp} values (pulling speed of k = 0.0005 nm ps^{-1}) 26
Figure S5. Correlation of W and ΔG_{exp} values (pulling speed of k = 0.005 nm ps^{-1}). 26
Figure S6. Correlation of W and ΔG_{exp} values (pulling speed of k = 0.002 nm ps^{-1}) 26
Figure S7. Correlation of W and ΔG_{exp} values (pulling speed of k = 0.001 nm ps^{-1}) 27
Figure S8. Correlation of W and ΔG_{exp} values (pulling speed of k = 0.0005 nm.ps^{-1}) 27

Table S1. Graph of F_{max} and W of twenty experimental complexes over time (pulling speed of k = 0.005 nm ps^{-1})

No.	ID	F_{max}	W
1	2W05	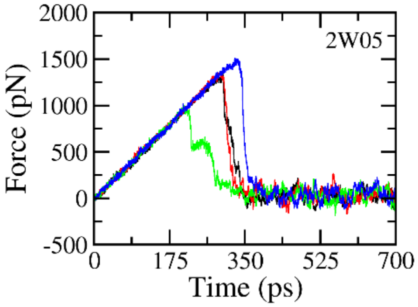	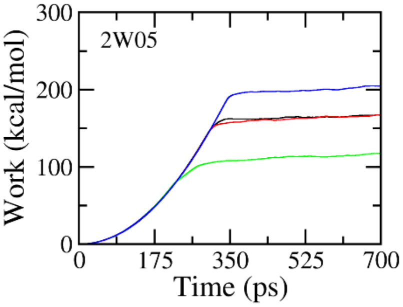
2	2W17	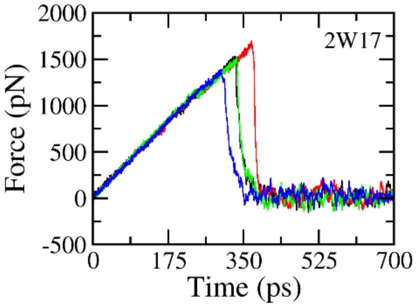	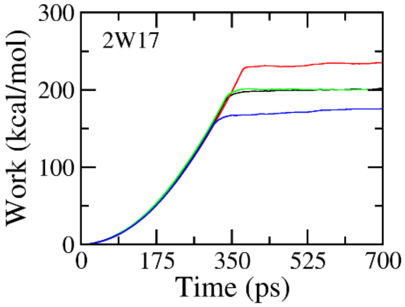
3	3NS9	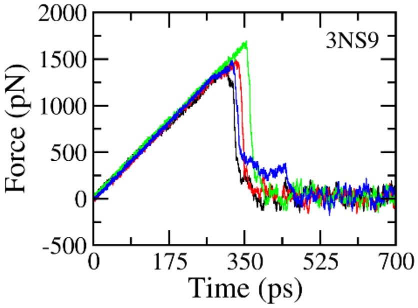	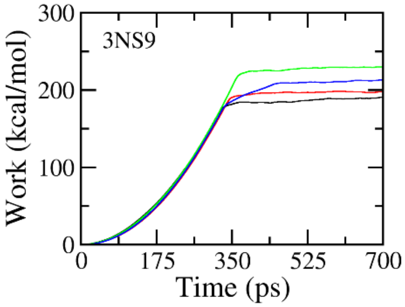
4	6GVA	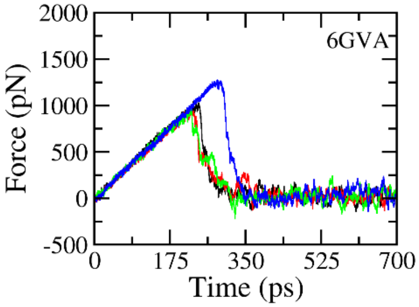	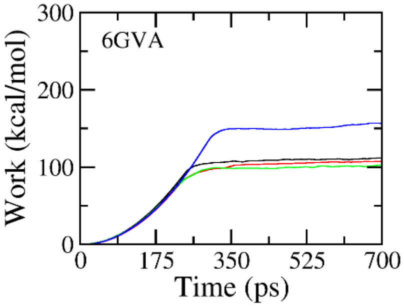

5 2R3J

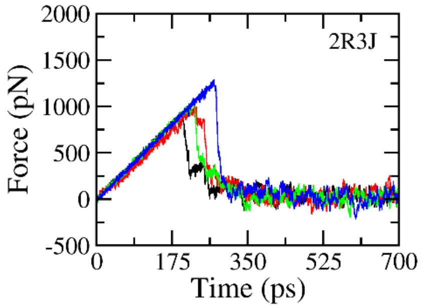

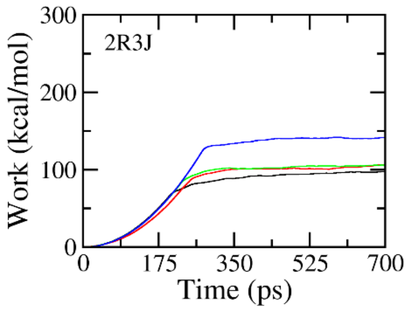

6 2VV9

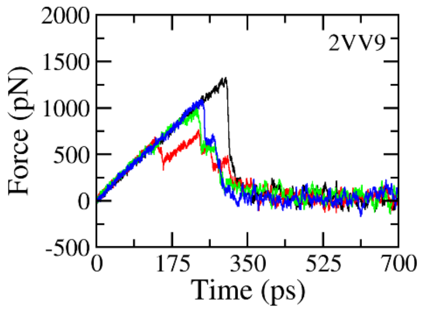

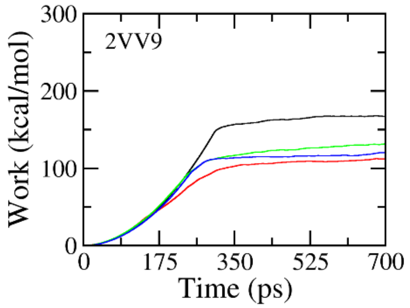

7 3PJ8

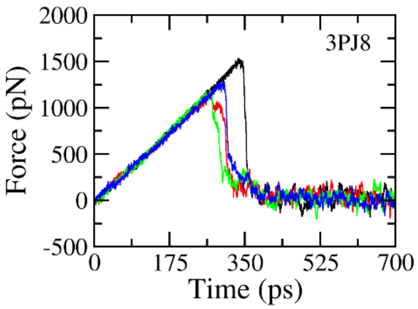

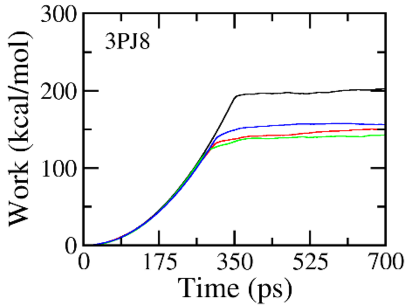

8 3S2P

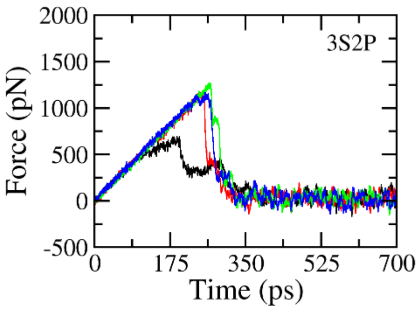

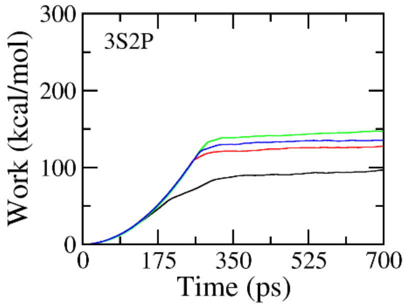

9 4RJ3

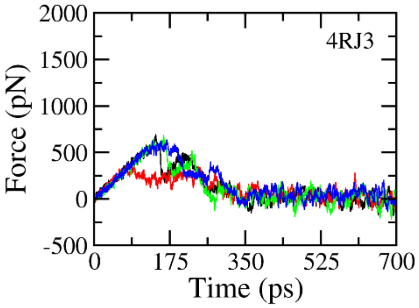

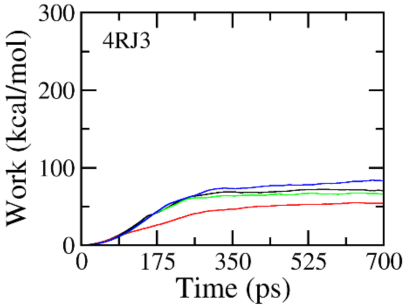

10 3PY0

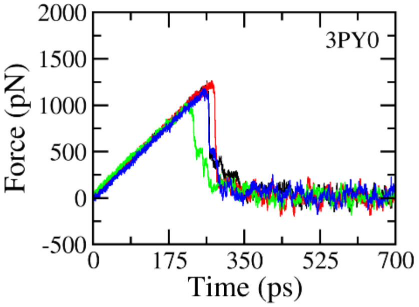

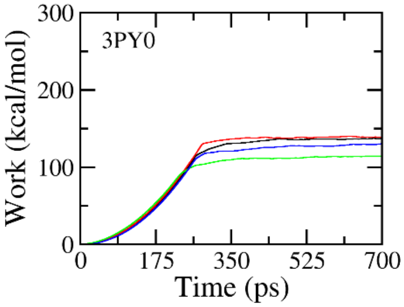

11 3DDQ

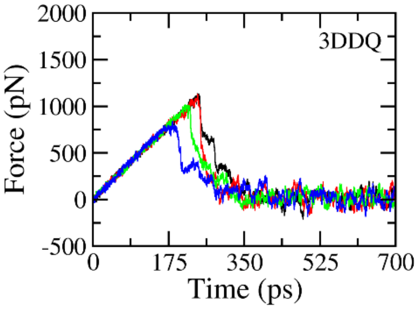

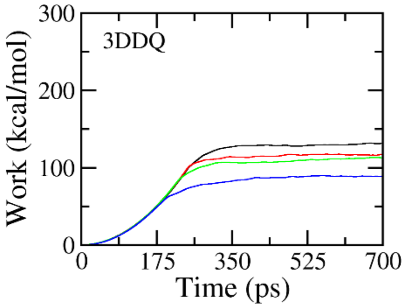

12 3EID

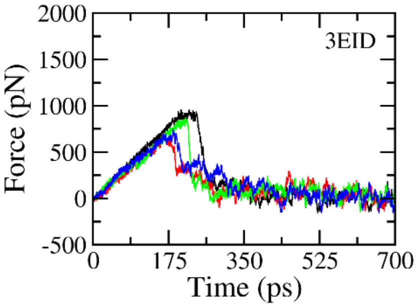

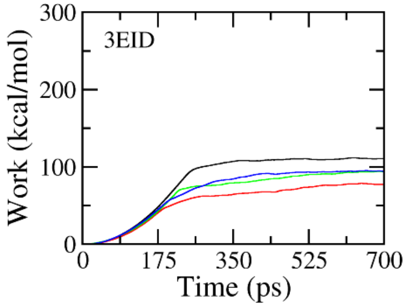

13 2R3I

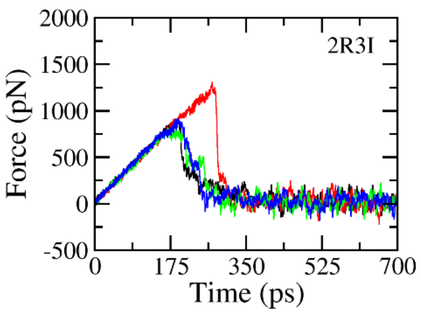

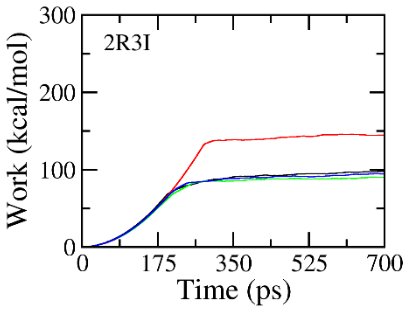

14 2I40

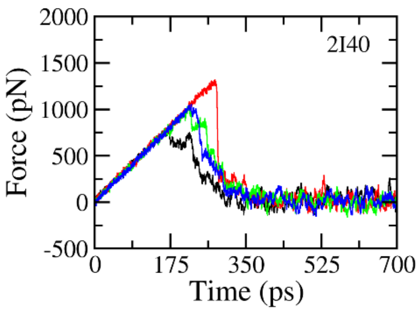

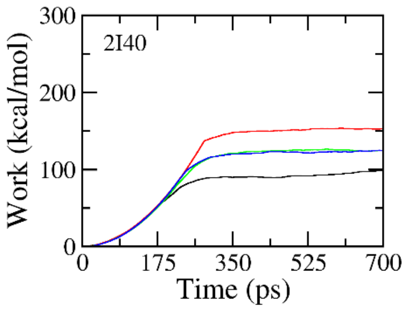

15 3LFS

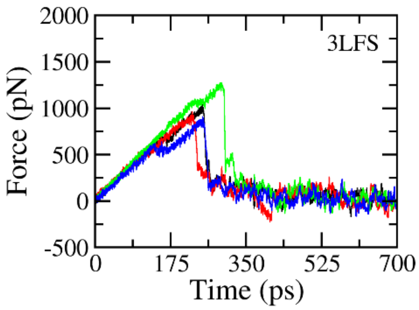

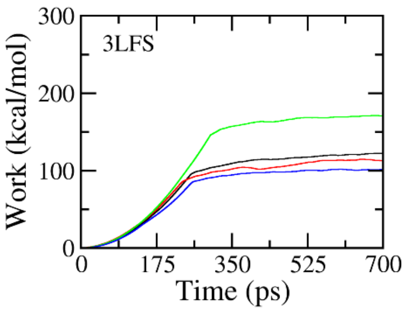

16 3PXY

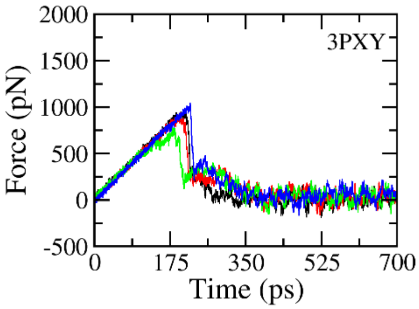

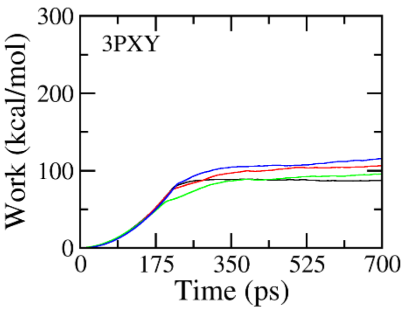

17	4CFX	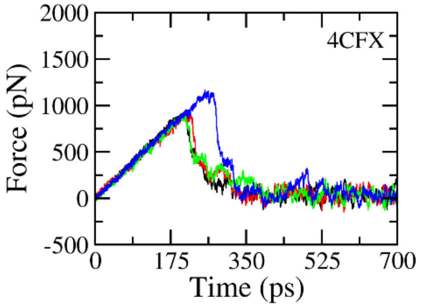	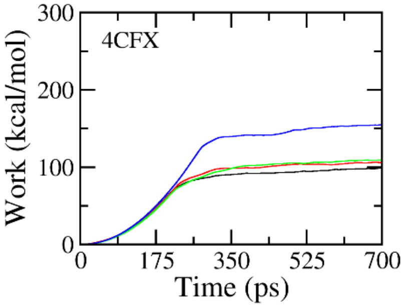
18	2R3H	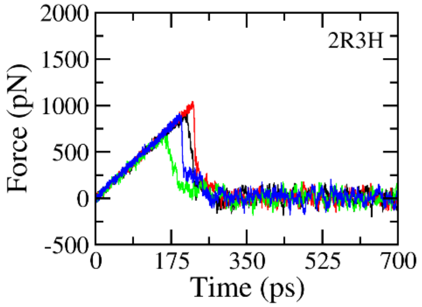	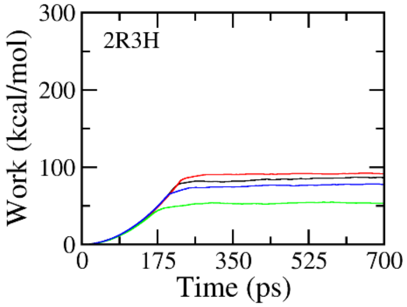
19	1V1K	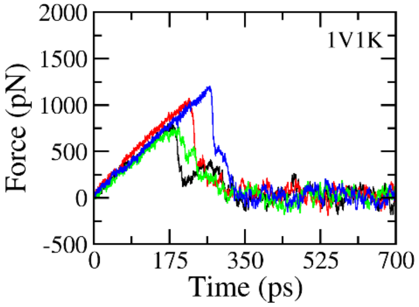	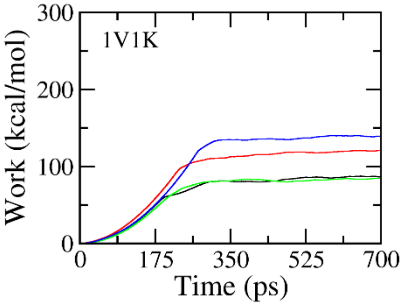
20	3MY5	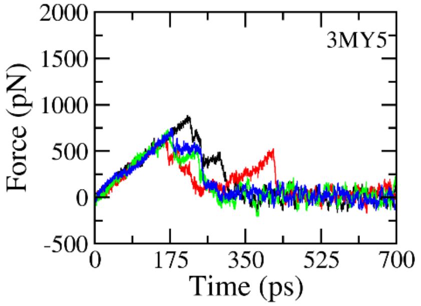	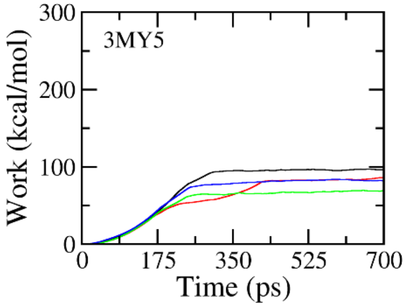

Table S2. Graph of F_{max} and W of twenty experimental complexes over time (pulling speed of k = 0.002 nm ps^{-1})

**No.**	**ID**	**F_{max}**	**W**

1 2W05

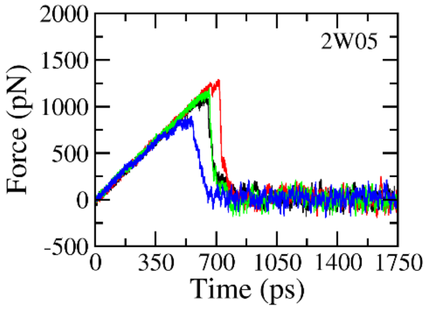

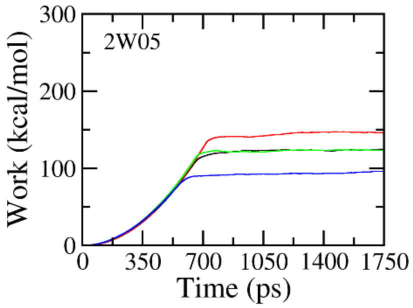

2 2W17

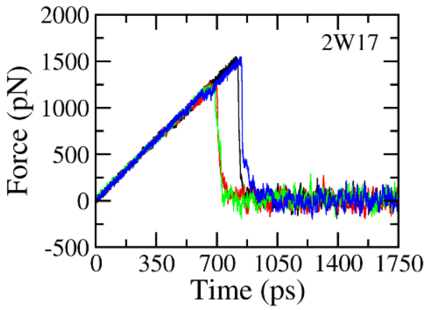

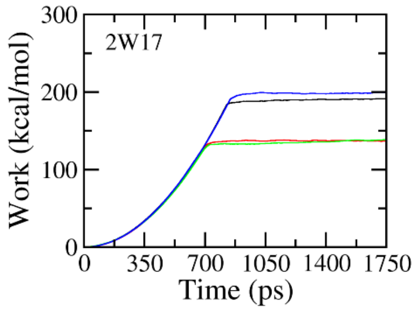

3 3NS9

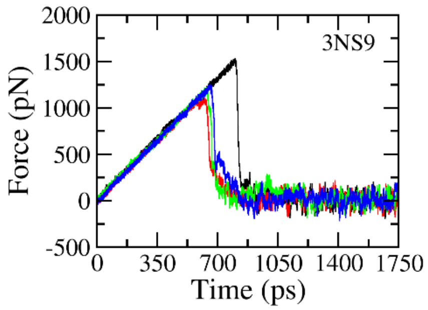

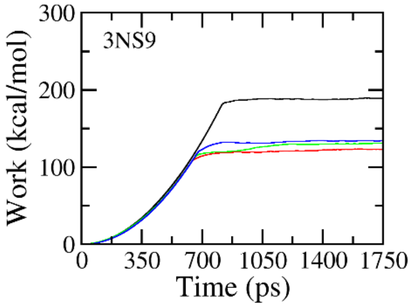

4 6GVA

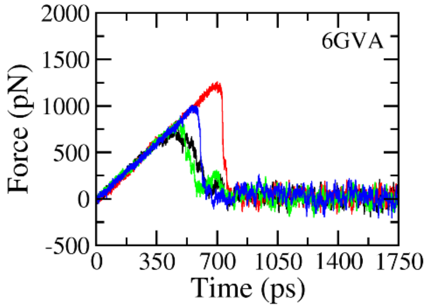

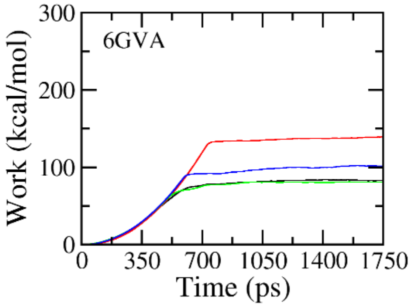

5 2R3J

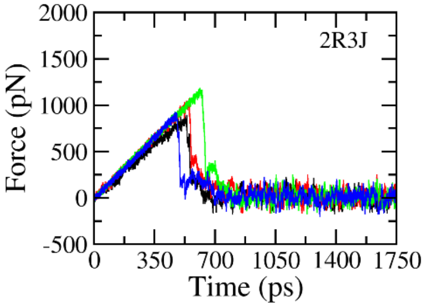

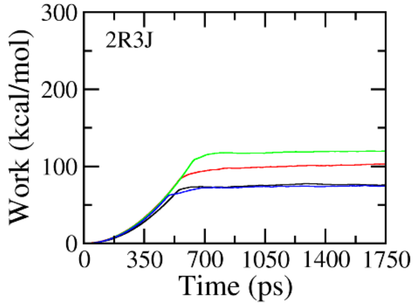

6 2VV9

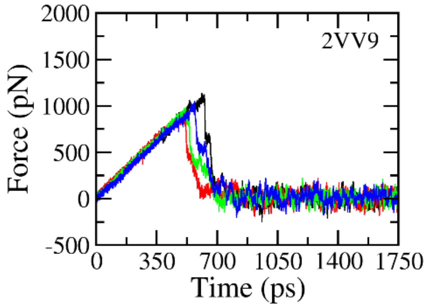

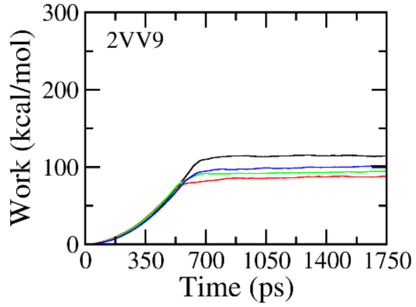

7 3PJ8

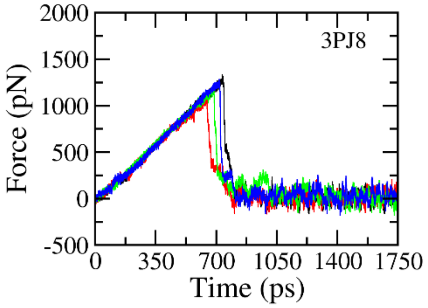

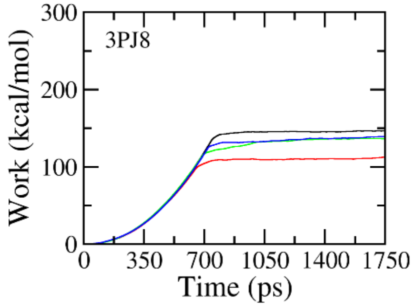

8 3S2P

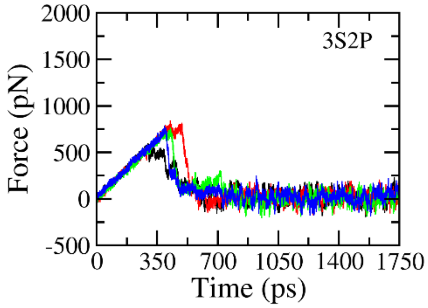

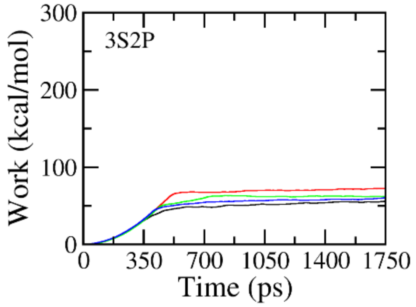

9 4RJ3

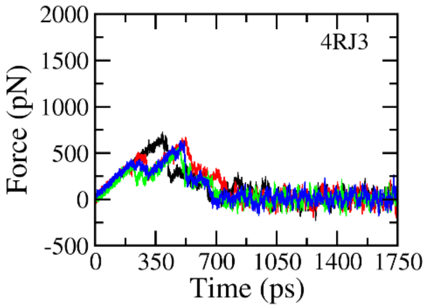

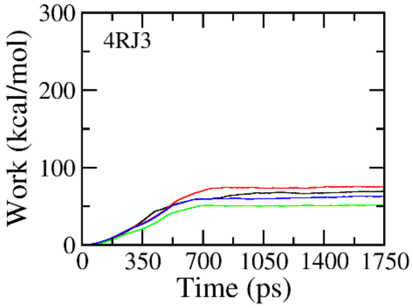

10 3PY0

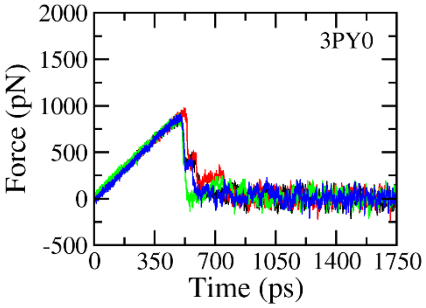

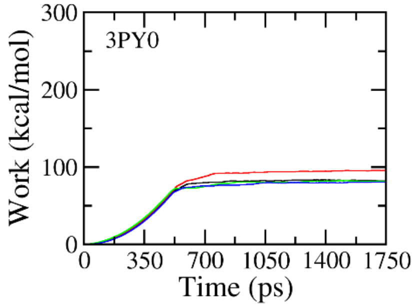

11 3DDQ

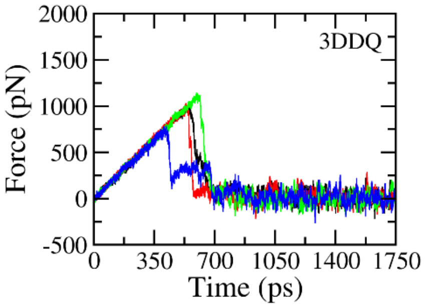

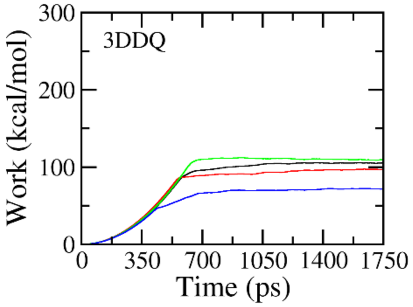

12 3EID

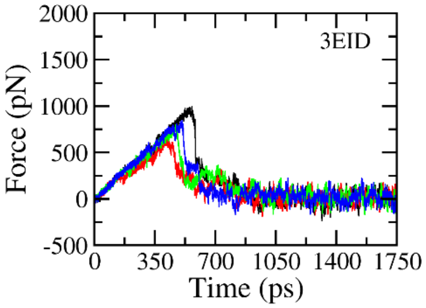

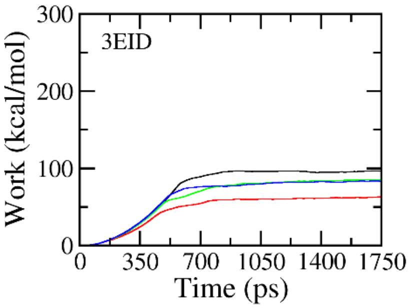

13 2R3I

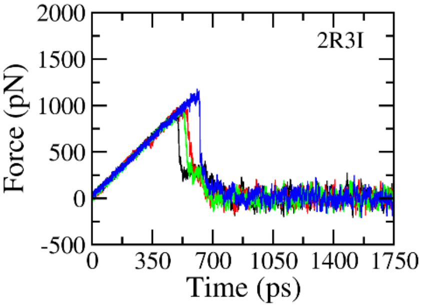

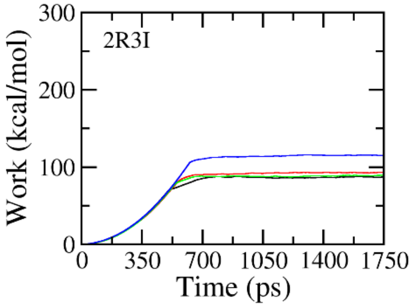

14 2I40

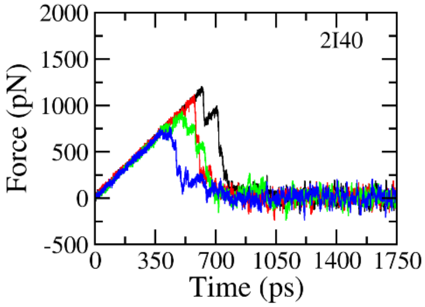

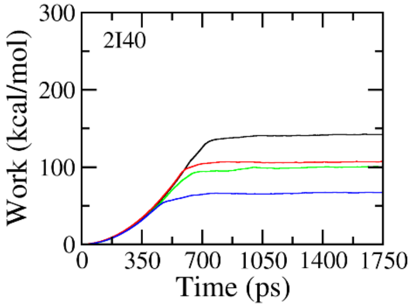

15 3LFS

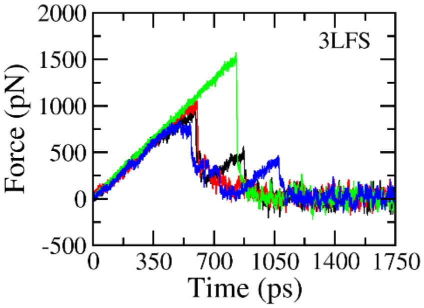

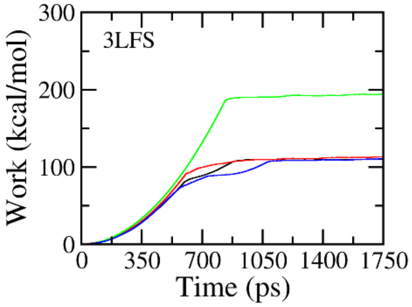

16 3PXY

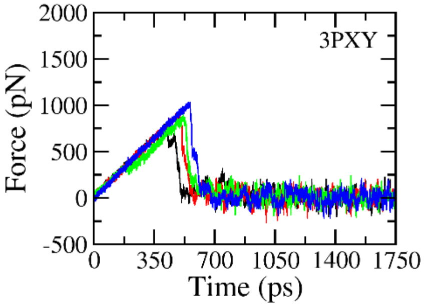

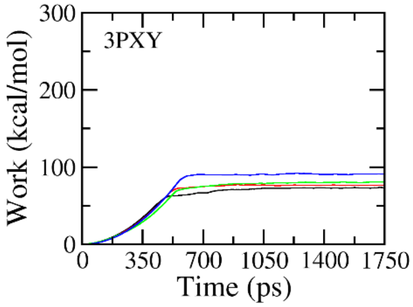

17	4CFX	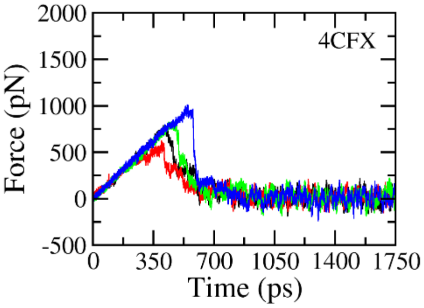	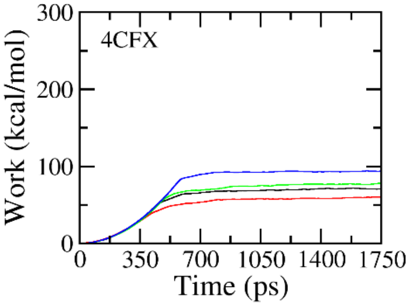
18	2R3H	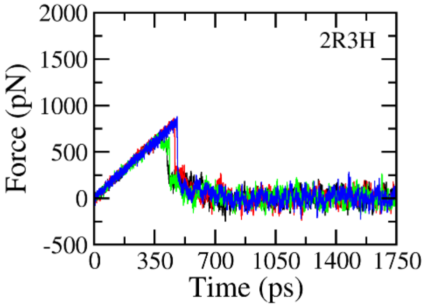	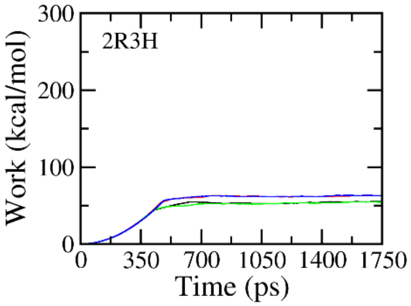
19	1V1K	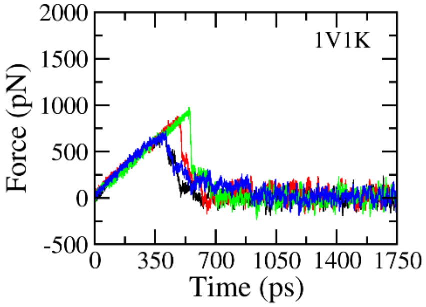	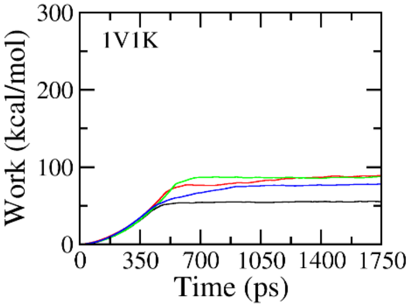
20	3MY5	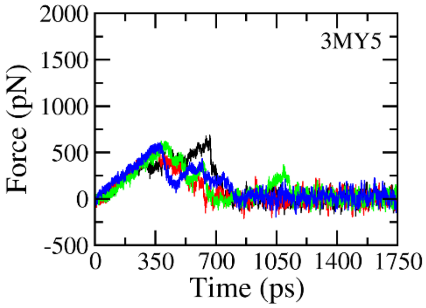	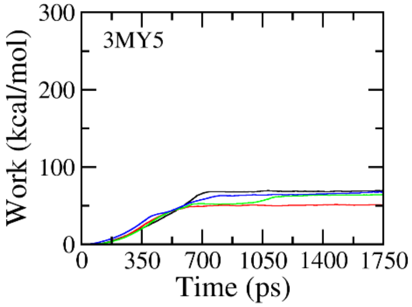

Table S3. Graph of F_{max} and W of twenty experimental complexes over time (pulling speed of k = 0.001 nm ps^{-1})

No.	ID	F_{max}	W

1 2W05

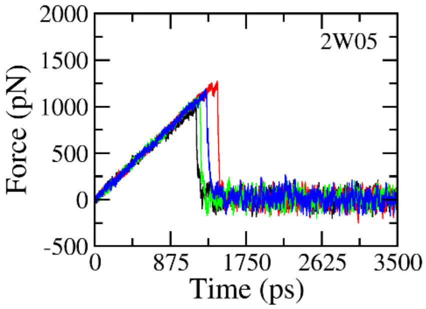

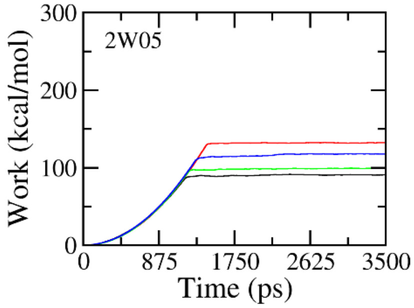

2 2W17

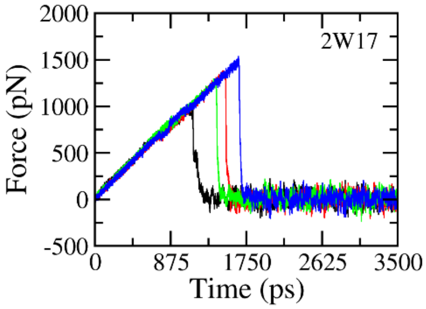

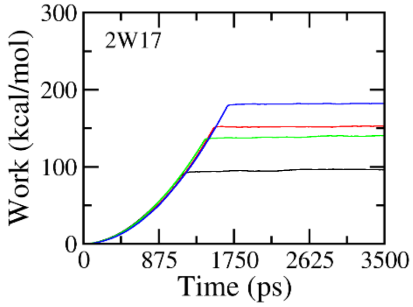

3 3NS9

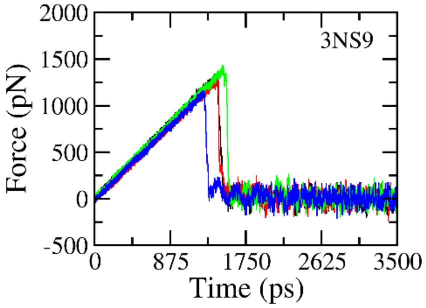

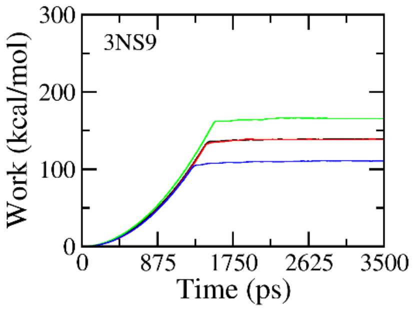

4 6GVA

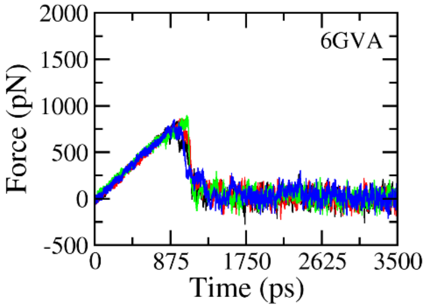

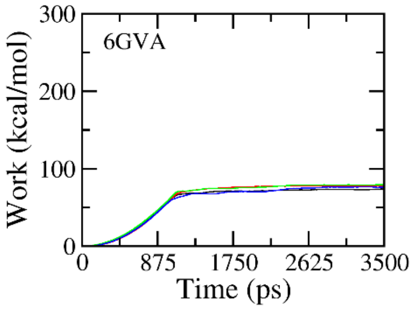

5 2R3J

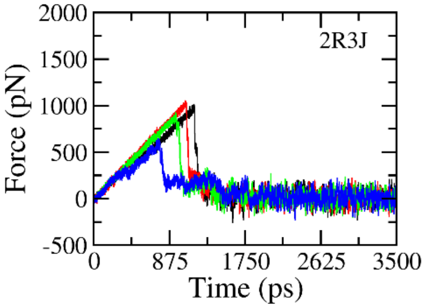

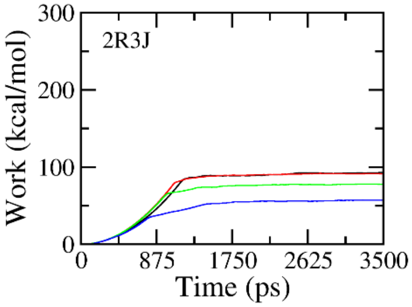

6 2VV9

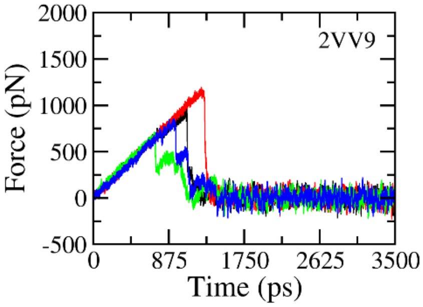

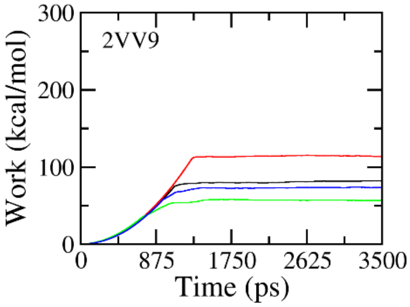

7 3PJ8

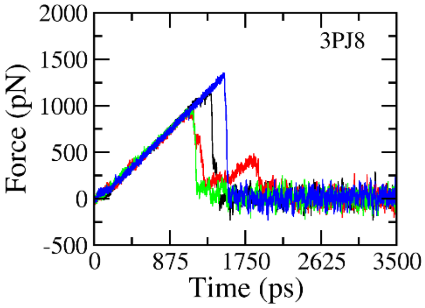

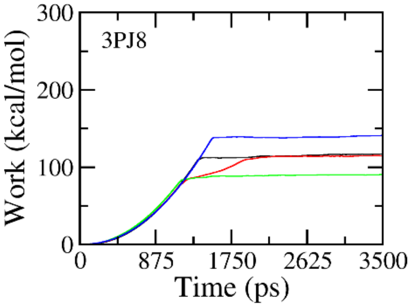

8 3S2P

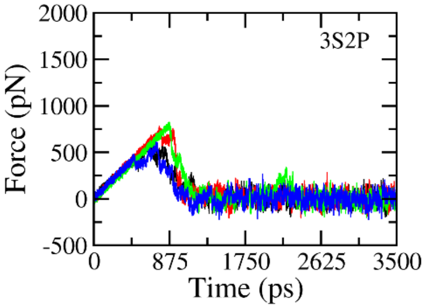

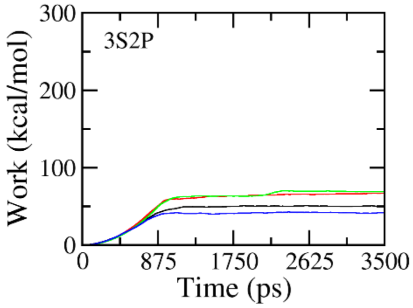

9	4RJ3	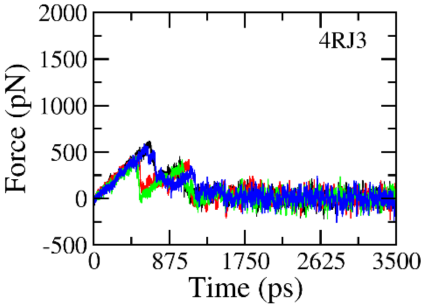	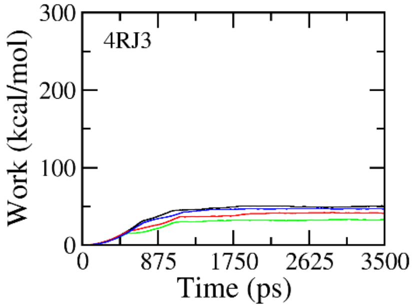
10	3PY0	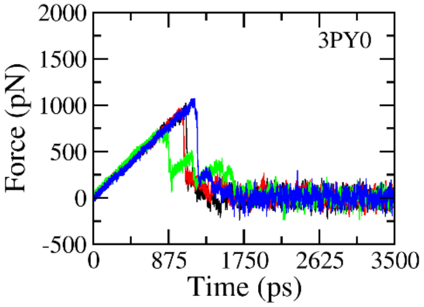	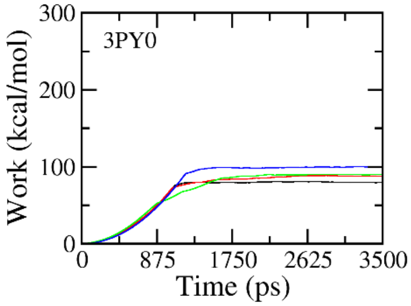
11	3DDQ	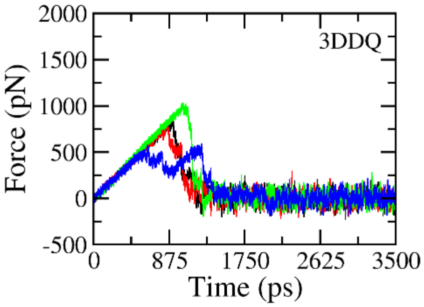	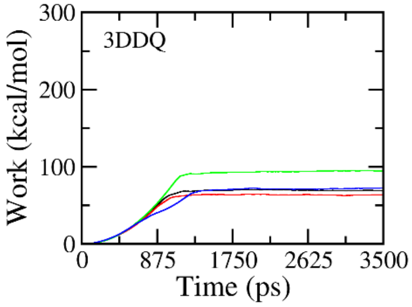
12	3EID	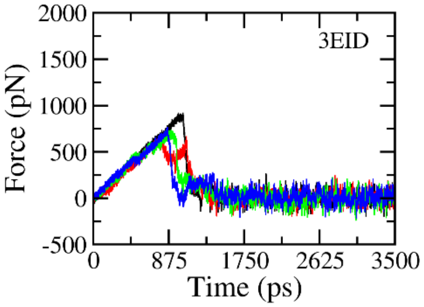	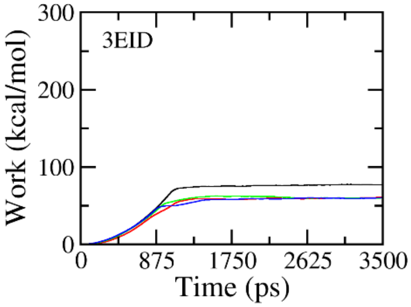

13 2R3I

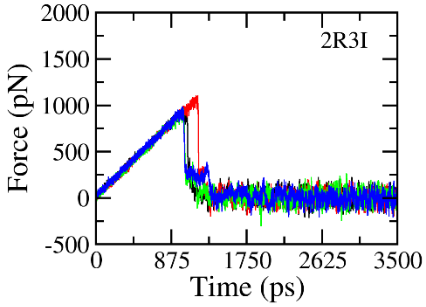

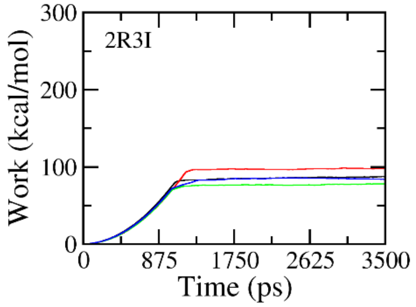

14 2I40

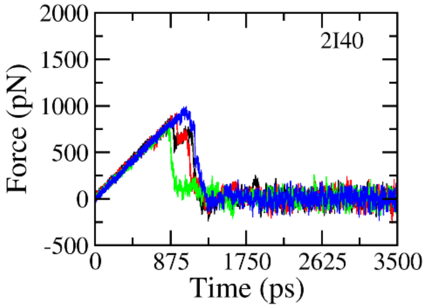

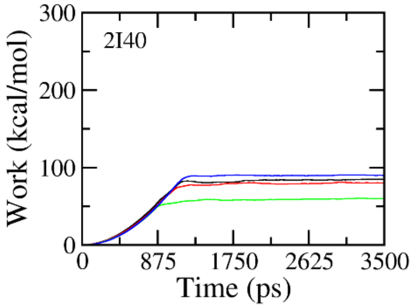

15 3LFS

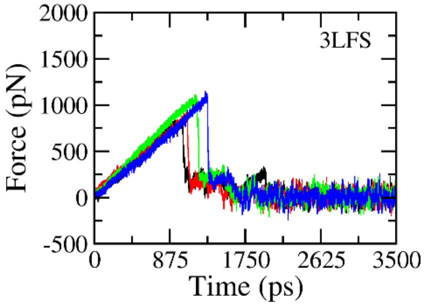

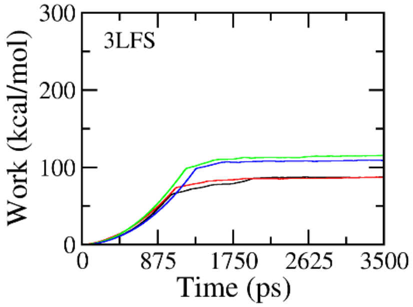

16 3PXY

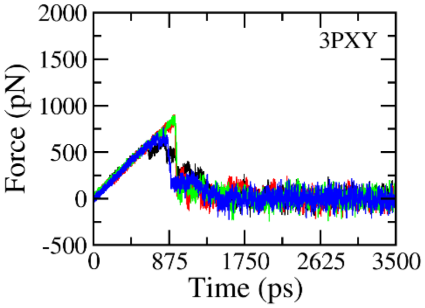

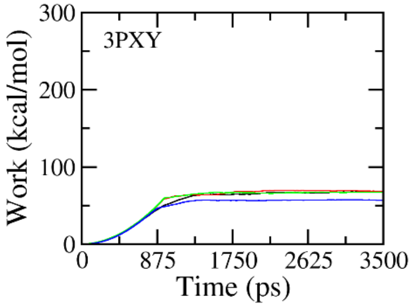

17	4CFX	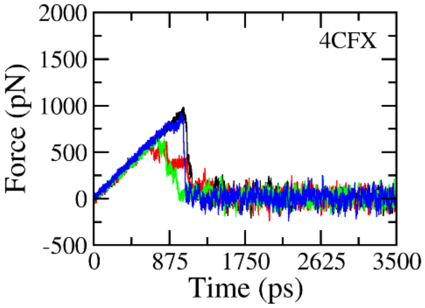	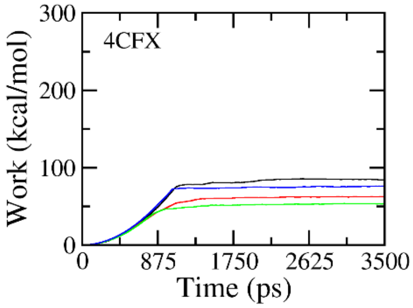
18	2R3H	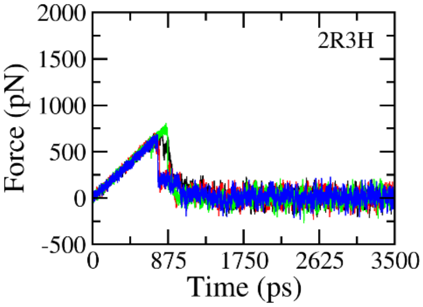	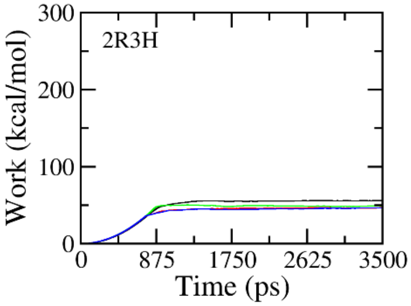
19	1V1K	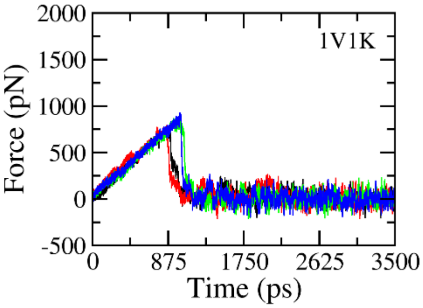	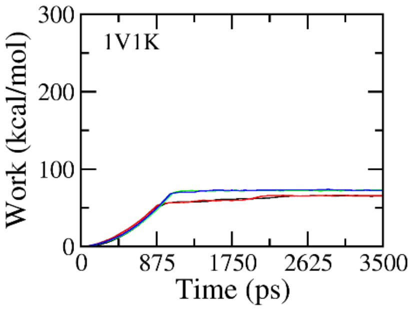
20	3MY5	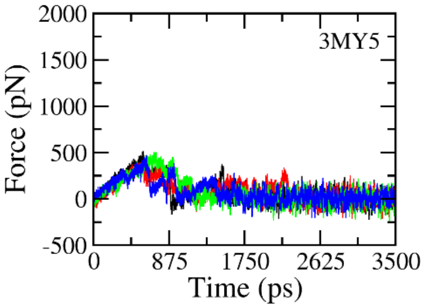	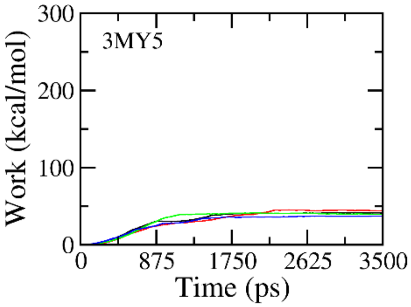

Table S4. Graph of F_{max} and W of twenty experimental complexes over time (pulling speed of k = 0.0005 nm ps^{-1})

No.	ID	F_{max}	W

1 2W05

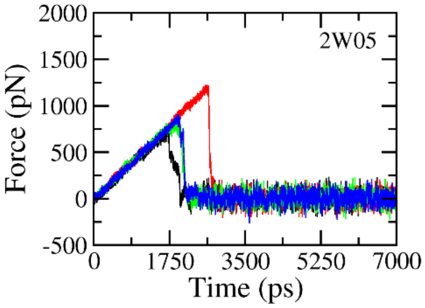

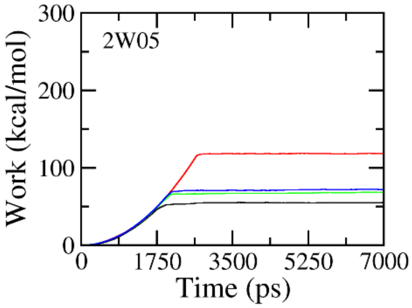

2 2W17

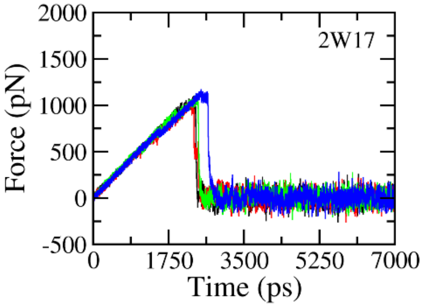

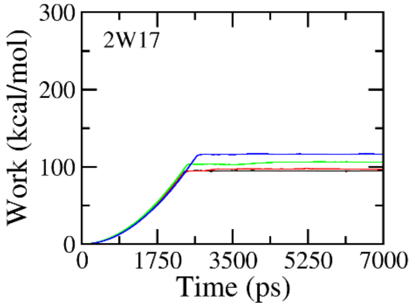

3 3NS9

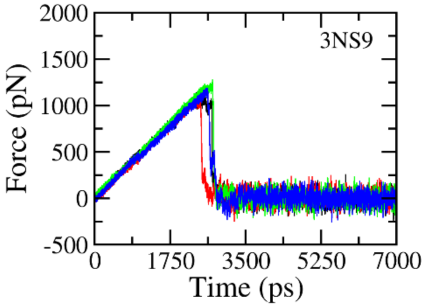

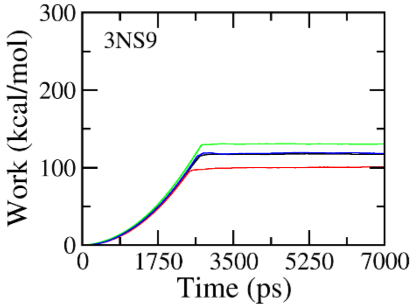

4 6GVA

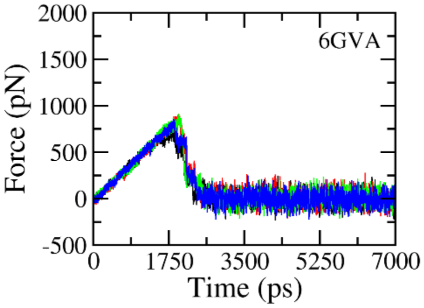

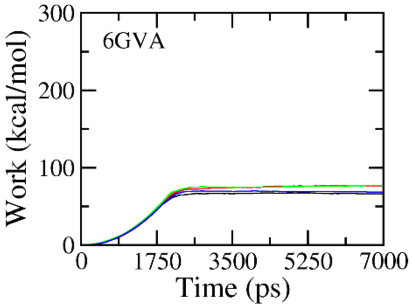

5 2R3J

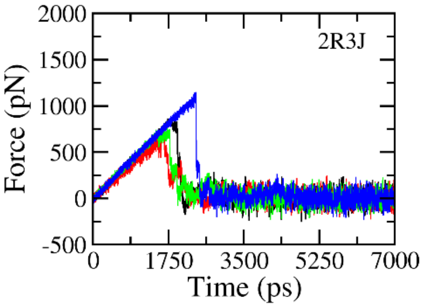

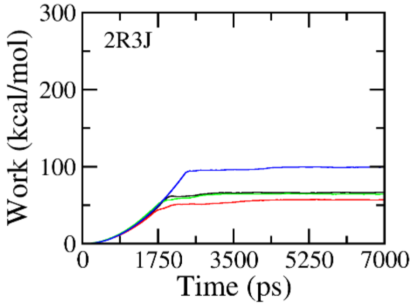

6 2VV9

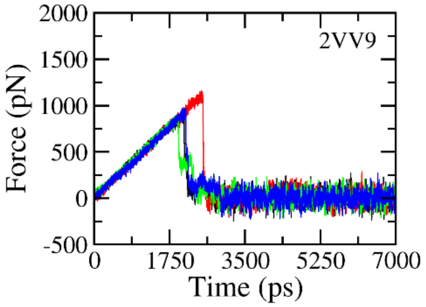

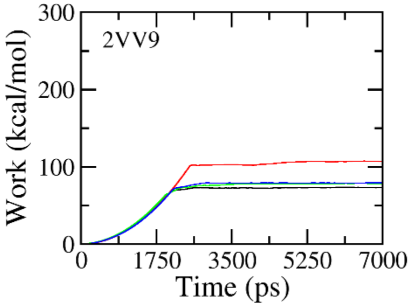

7 3PJ8

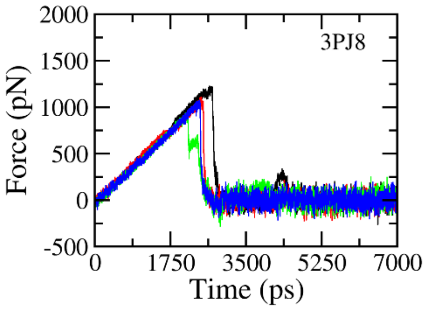

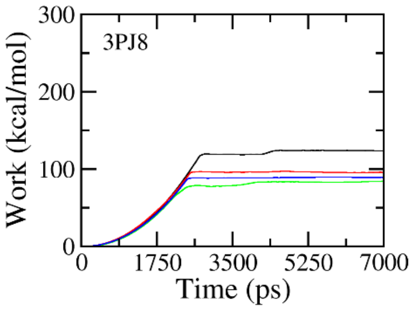

8 3S2P

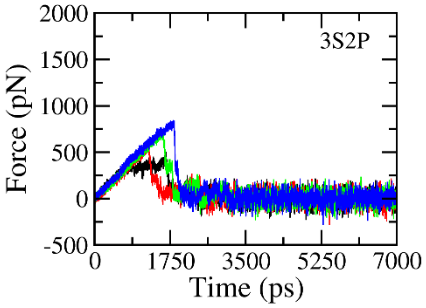

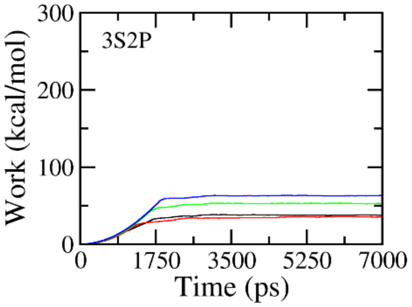

9	4RJ3	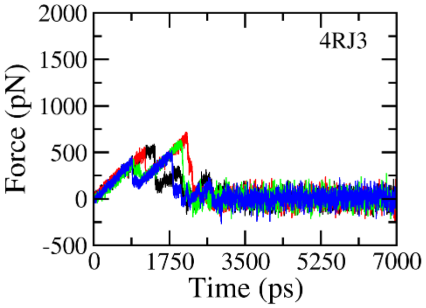	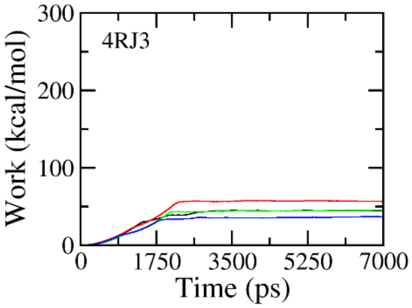
10	3PY0	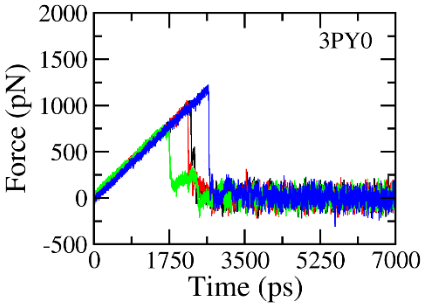	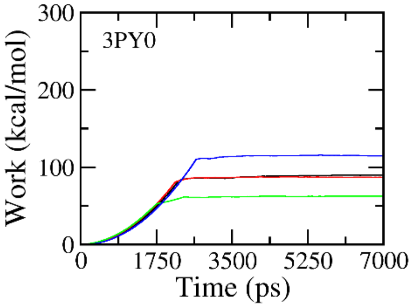
11	3DDQ	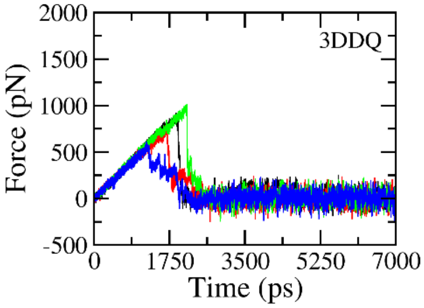	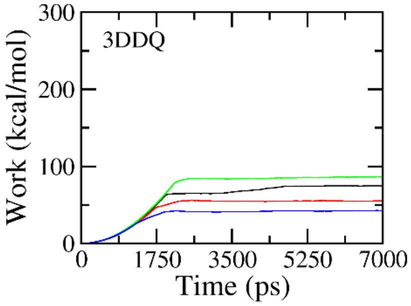
12	3EID	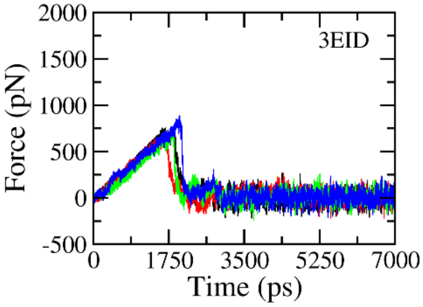	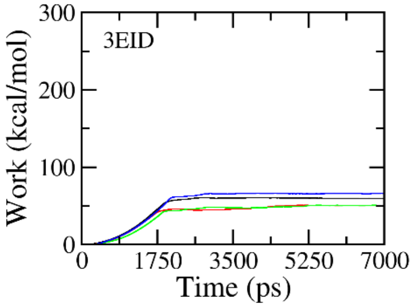

13 2R3I

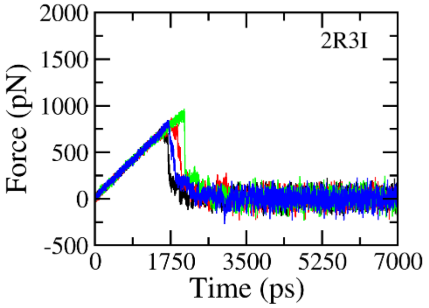

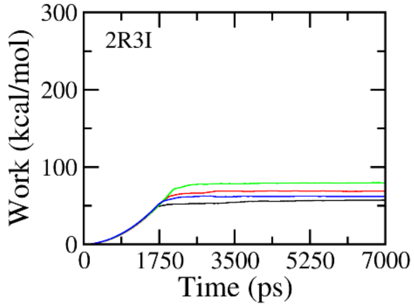

14 2I40

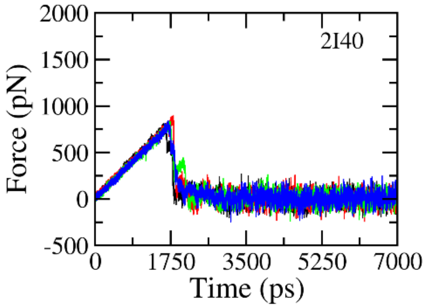

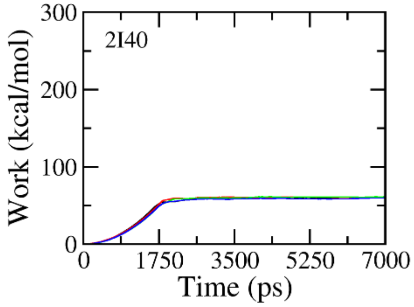

15 3LFS

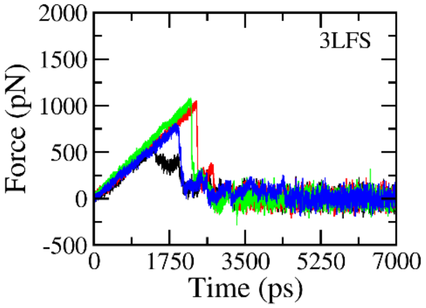

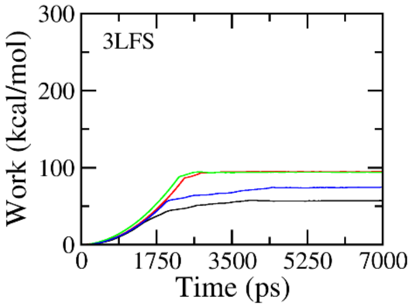

16 3PXY

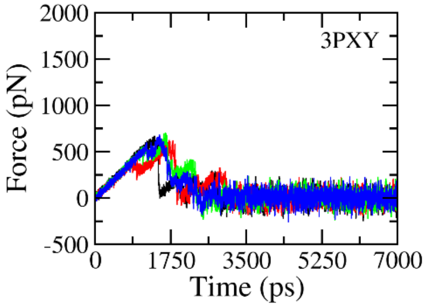

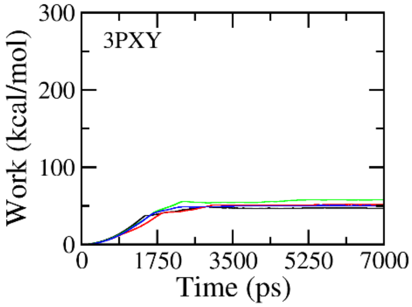

17	4CFX	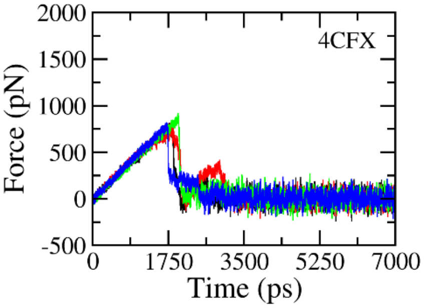	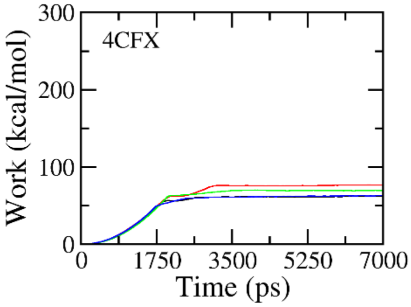
18	2R3H	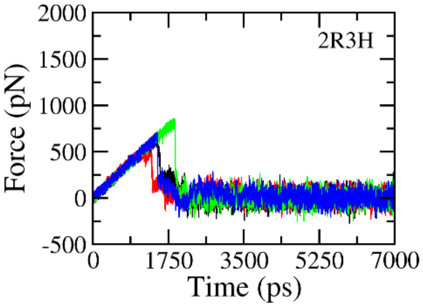	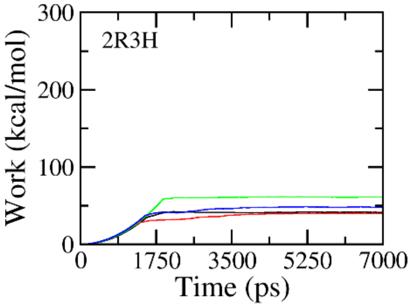
19	1V1K	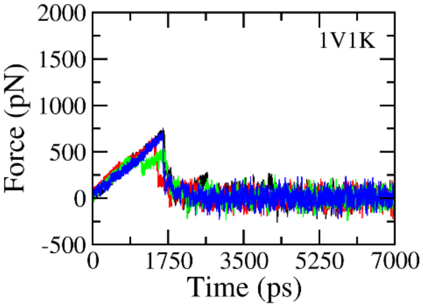	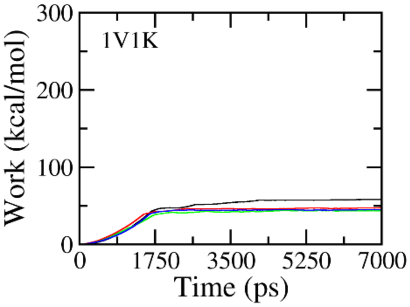
20	3MY5	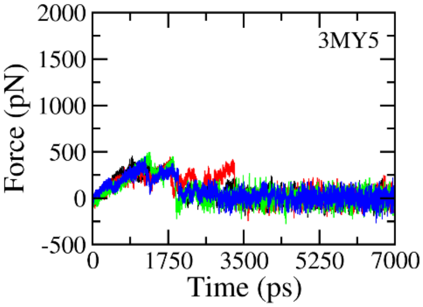	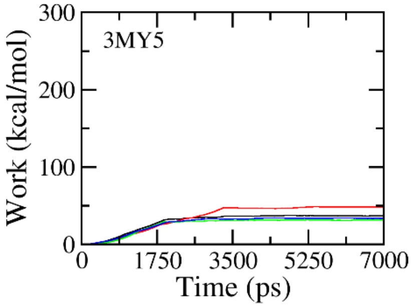

Table S5. FPL results of maximum pulling force (F_{max}) of 20 experimental structures

No.	PDB ID	ΔGexp (kcal/mol)	Fmax (pN) k = 0.005	Fmax (pN) k = 0.002	Fmax (pN) k = 0.001	Fmax (pN) k = 0.0005
1	2W05	-12.76	1300.8 ± 92.8	1122.8 ± 72.2	1151.6 ± 43.6	929.9 ± 89.7
2	2W17	-12.34	1529.6 ± 55.4	1404.0 ± 71.5	1305.0 ± 95.8	1110.1 ± 21.2
3	3NS9	-12.09	1527.2 ± 48.2	1275.3 ± 78.2	1308.8 ± 48.9	1173.2 ± 35.0

4	6GVA	-11.66	1066.6 ± 61.0	986.0 ± 84.9	860.4 ± 11.4	866.7 ± 24.6
5	2R3J	-11.35	1078.2 ± 64.4	1012.7 ± 58.9	907.8 ± 79.5	878.7 ± 81.6
6	2VV9	-11.02	1044.9 ± 100.7	1015.4 ± 41.2	930.1 ± 88.9	992.2 ± 50.8
7	3PJ8	-10.49	1289.8 ± 71.5	1225.1 ± 45.5	1122.5 ± 78.8	1090.5 ± 48.9
8	3S2P	-10.17	1072.9 ± 111.8	739.8 ± 45.9	705.6 ± 47.4	663.2 ± 65.0
9	4RJ3	-9.97	592.6 ± 66.6	647.6 ± 28.4	531.4 ± 35.4	617.9 ± 34.4
10	3PY0	-9.77	1180.7 ± 49.1	928.6 ± 14.6	952.2 ± 62.3	1040.0 ± 75.9
11	3DDQ	-9.47	1026.3 ± 60.5	986.8 ± 65.1	826.6 ± 78.6	828.6 ± 76.9
12	3EID	-9.00	809.0 ± 55.9	825.7 ± 57.9	768.9 ± 46.4	774.4 ± 38.4
13	2R3I	-8.51	986.5 ± 94.8	1028.8 ± 43.6	1005.7 ± 28.5	866.3 ± 37.2
14	2I40	-8.23	1051.3 ± 92.0	1007.5 ± 83.2	905.0 ± 31.0	846.7 ± 13.9
15	3LFS	-7.95	1044.8 ± 70.1	1108.1 ± 138.0	1010.5 ± 60.9	894.4 ± 91.9
16	3PXY	-7.42	930.0 ± 47.3	912.3 ± 40.3	801.5 ± 50.7	672.5 ± 13.1
17	4CFX	-7.18	975.7 ± 55.5	809.3 ± 68.9	800.9 ± 76.3	840.5 ± 23.4
18	2R3H	-6.66	899.5 ± 59.9	797.7 ± 37.7	734.4 ± 25.0	707.5 ± 52.1
19	1V1K	-6.32	955.8 ± 93.3	827.8 ± 51.9	838.4 ± 30.0	665.1 ± 41.9
20	3MY5	-5.94	758.2 ± 37.9	619.5 ± 22.2	473.7 ± 19.4	460.1 ± 9.9

*Note: Pulling speed of k in nm.ps^{-1}. Error is the standard error of mean.

Table S6. Graph of F_{max} and W of three top-lead compounds over time (pulling speed of k = 0.005 nm ps^{-1})

**No.**	**CID ID**	F_{max}	**W**
1	636885	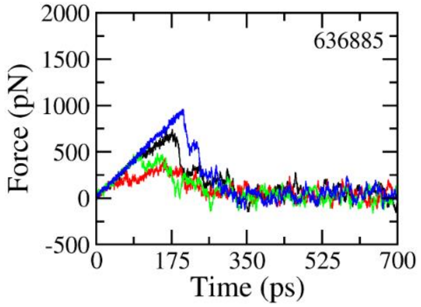	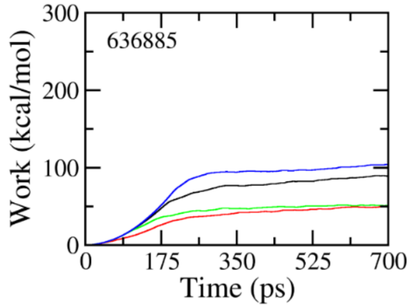
2	46184320	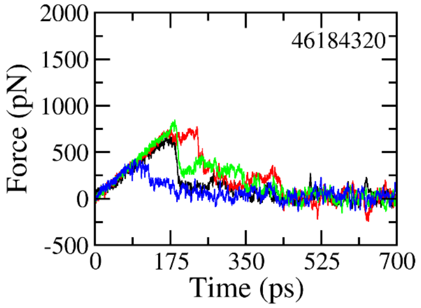	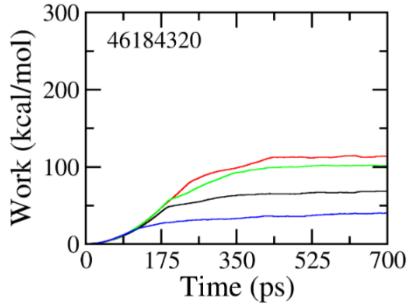

3 101691758

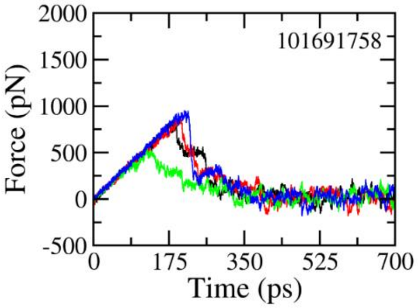

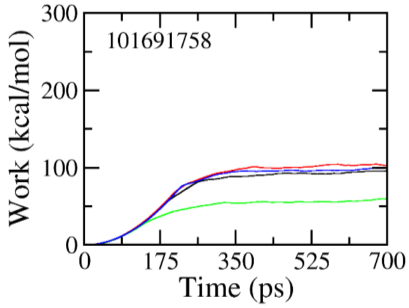

Table S7. List of CDK2 residues forming SC/HB contacts to the three top-lead compounds

CID 636885

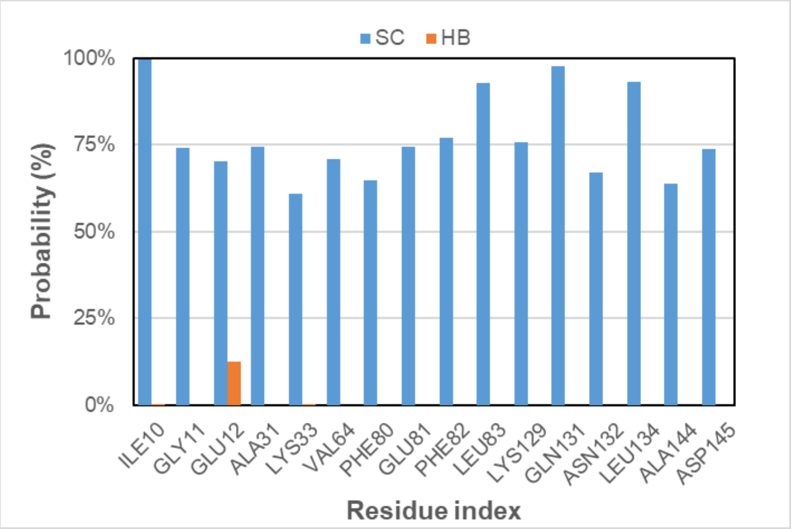

CID 46184320

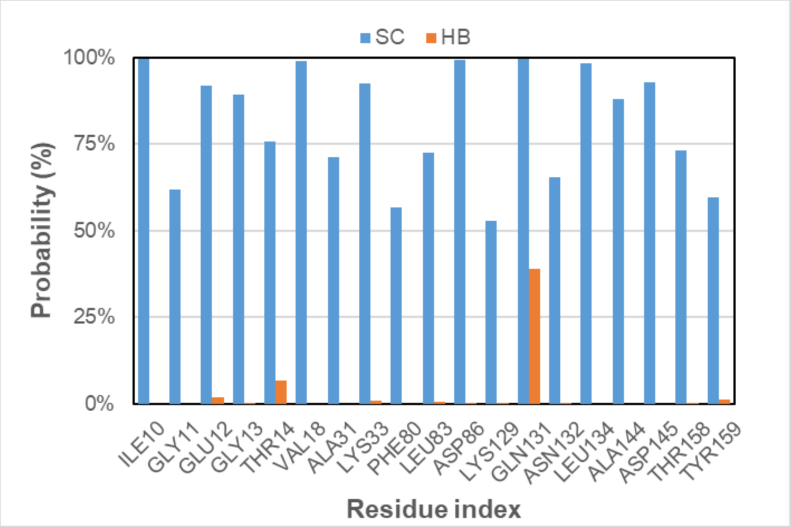

CID 101691758

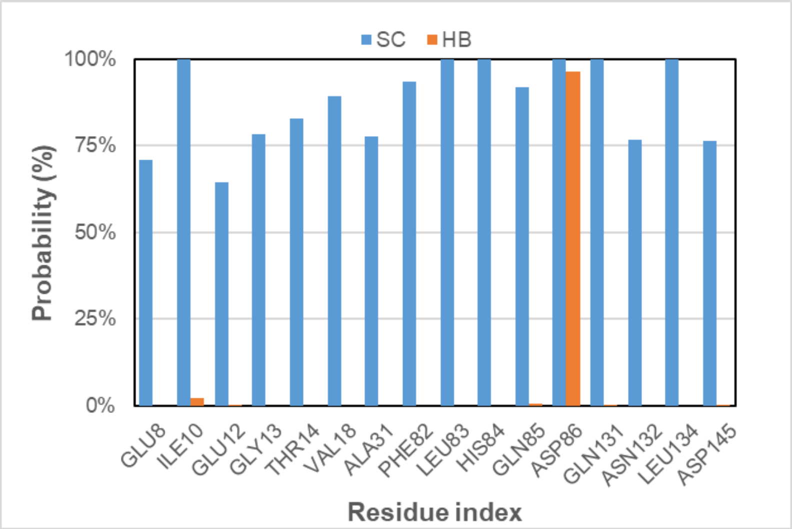

Total

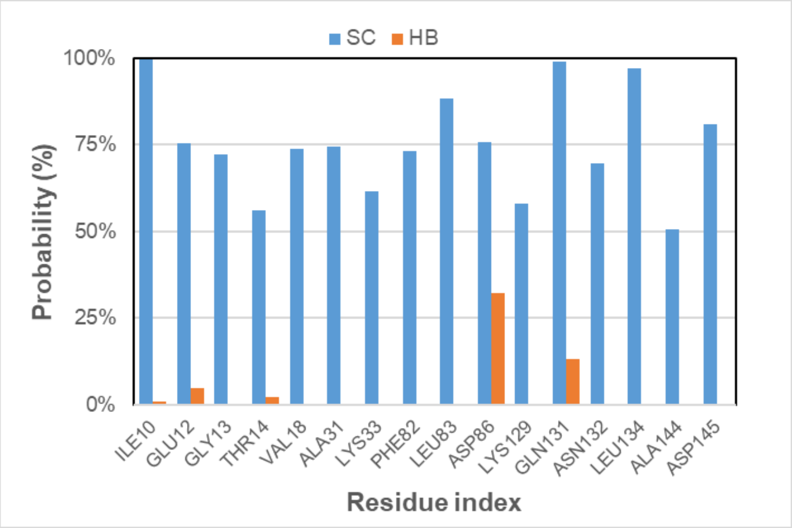

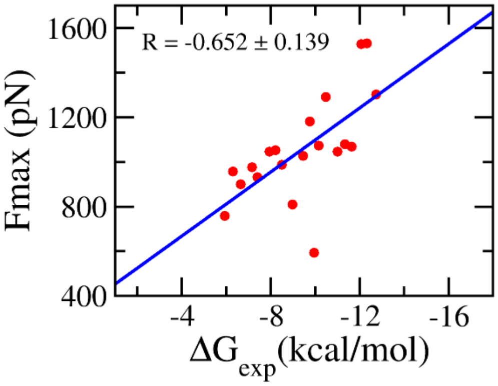

Figure S1. Correlation of F_{max} and ΔG_{exp} values (pulling speed of k = 0.005 nm ps^{-1})

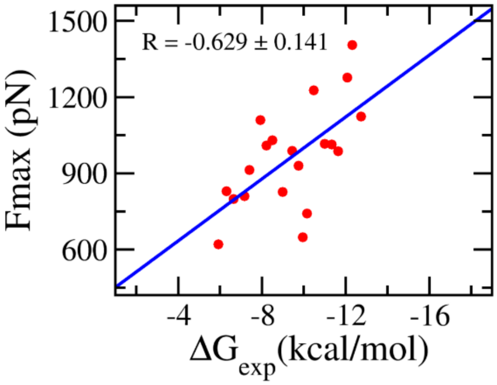

Figure S2. Correlation of F_{max} and ΔG_{exp} values (pulling speed of k = 0.002 nm ps^{-1})

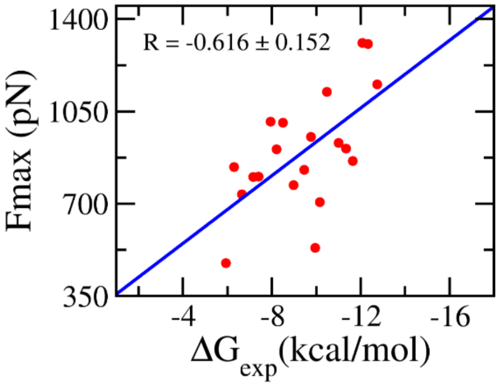

Figure S3. Correlation of F_{max} and ΔG_{exp} values (pulling speed of k = 0.001 nm ps^{-1})

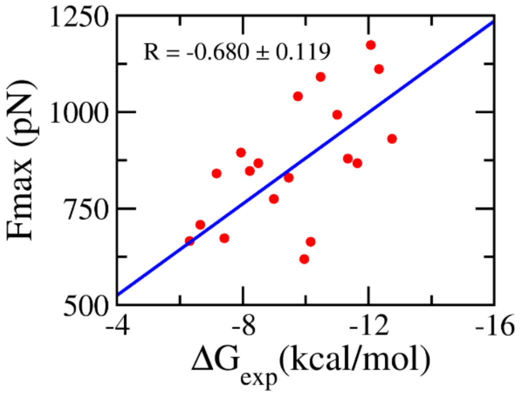

Figure S4. Correlation of F_{max} and ΔG_{exp} values (pulling speed of k = 0.0005 nm ps^{-1})

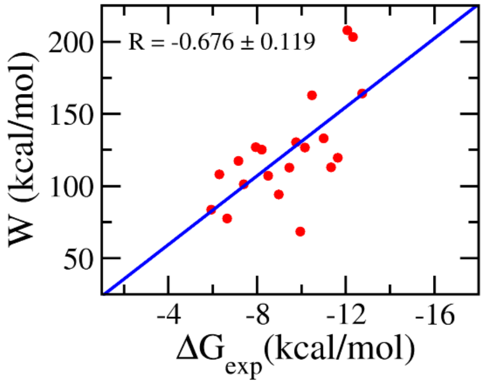

Figure S5. Correlation of W and ΔG_{exp} values (pulling speed of k = 0.005 nm ps^{-1}).

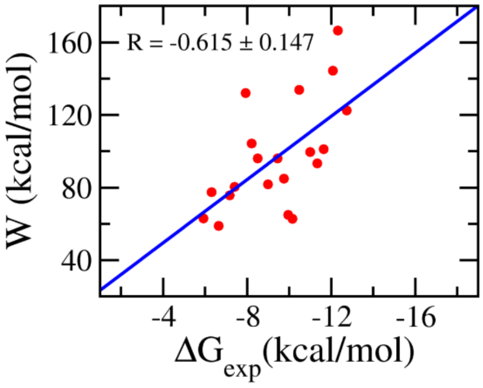

Figure S6. Correlation of W and ΔG_{exp} values (pulling speed of k = 0.002 nm ps^{-1})

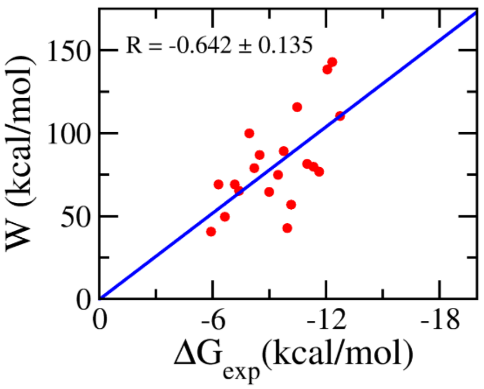

Figure S7. Correlation of W and ΔG_{exp} values (pulling speed of k = 0.001 nm ps^{-1})

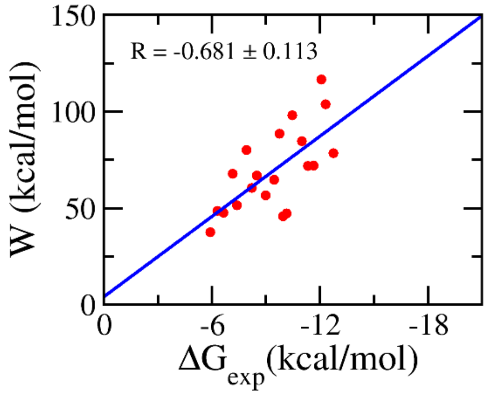

Figure S8. Correlation of W and ΔG_{exp} values (pulling speed of k = 0.0005 nm ps^{-1})
